# Novel Indole-Containing Hybrids Derived from Millepachine: Synthesis, Biological Evaluation and Antitumor Mechanism Study

**DOI:** 10.3390/molecules28031481

**Published:** 2023-02-03

**Authors:** Baoxia Liang, Qing Zou, Lintao Yu, Yali Wang, Jun Yan, Baiqi Huang

**Affiliations:** 1 The School of Food Science and Biology, Guangdong Polytechnic of Science and Trade, Guangzhou 510430, China; 2 Second Clinical Medical College, Guangzhou University of Chinese Medicine, Guangzhou 510120, China; 3 BGI Infection Pharmaceutical Technology, BGI-Shenzhen, Shenzhen 518083, China

**Keywords:** tubulin polymerization inhibitors, millepachine, antitumor

## Abstract

Millepachine, a bioactive natural product isolated from the seeds of Millettia pachycarpa, is reported to display potential antitumor activity. In this study, novel indole-containing hybrids derived from millepachine were designed, synthesized and evaluated for their antitumor activities. Among all the compounds, compound **14b** exhibited the most potent cytotoxic activity against five kinds of human cancer cell lines, with IC_50_ values ranging from 0.022 to 0.074 μM, making it almost 100 times more active than millepachine. Valuable structure–activity relationships (SARs) were obtained. Furthermore, the mechanism studies showed that compound **14b** induced cell-cycle arrest at the G2/M phase by inhibiting tubulin polymerization and further induced cell apoptosis through reactive oxygen species (ROS) accumulation and mitochondrial membrane potential (MMP) collapse. In addition, the low cytotoxicity toward normal human cells and equivalent sensitivity towards drug-resistant cells of compound **14b** highlighted its potential for the development of antitumor drugs.

## 1. Introduction

Natural products (NPs) and their structural analogues are historically valuable sources of lead compounds for drug discovery, especially for cancer and infectious diseases [1]. However, the investigation of NPs for drug discovery is hampered by various inevitable difficulties, such as the low amount of NPs available for pharmacological evaluation, the limited availability of natural resources, the difficulty of total chemical synthesis, and the relatively low bioavailability in NPs [2,3]. Therefore, searching for small molecular compounds based on the structures of natural products is an important strategy for drug discovery [4]. The high structural diversity and various bioactivities of NPs provide diverse scaffolds for NP-based drug discovery.

Microtubules, which consist of α- and β-tubulin, are dynamic cytoskeleton components involved in a range of intracellular processes, such as substance transportation, beating of cilia and flagella, cell division and mitosis [5]. Tubulin inhibitors that interfere with the dynamic equilibrium of polymerization and depolymerization are also named antimitotic agents, and they not only perturb mitosis but also arrest cells during the interphase [6]. The prominent role of tubulin in cell division makes it an effective target for antitumor drugs, resulting in tremendous efforts in drug discovery targeting the tubulin–microtubule system [7,8].

Millepachine is a bioactive natural product isolated from the seeds of Millettia pachycarpa that is reported to have potential antitumor activity at the micromole level in cell-based evaluations [9]. On the basis of these reports, various attempts have been undertaken to obtain diverse millepachine-derived compounds, aiming to increase its antitumor activity [10,11,12]. Most mechanism studies demonstrated that millepachine exerted anticancer activity as a tubulin polymerization inhibitor that disturbed mitotic spindle formation and causes cell-cycle arrest at the G2/M phase, followed by apoptosis [13,14].

The indole moiety is one of the most broadly N-heterocyclic frameworks occurring in natural products and in the area of medicinal chemistry [15]. The interesting molecular architecture and wide spectrum of pharmacological activities of indole make it an important pharmacophore present in various drugs. Moreover, indole and its derivatives are well-known, privileged scaffolds that have been used in the discovery and development of many antitumor drug molecules [16,17]. Indole derivatives, such as tubulin inhibitors, comprise a vast area in chemotherapeutic drug discovery [18,19]. They include numerous natural indole-containing derivatives, such as vinca alkaloid, moroidin and diazionamide A, and synthetic indole-based compounds, such as indibulin, rosabulin and MKC-1, have been reported as tubulin inhibitors with prominent positions in clinical trials in anticancer drug development [20].

**Figure 1:**
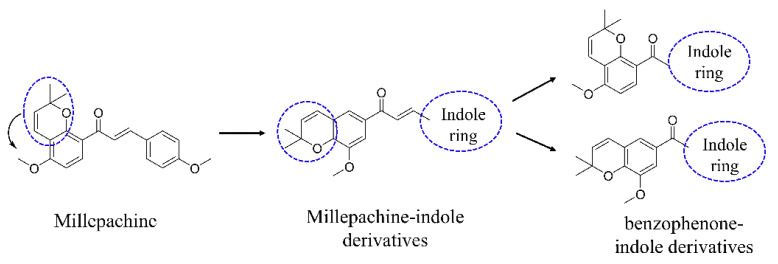
The design strategy for indole-containing hybrids derived from millepachine.

In recent years, our group has been committed to the design and development of indole-containing antitumor agents that act as tubulin polymerization inhibitors [21,22,23,24,25,26]. In this study, as shown in Figure 1, starting from the structure of millepachine, we moved the position of the 2,2-dimethylbenzopyran group and introduced the indole ring to obtain millepachine-indole derivatives. Then, by replacing the α,β-unsaturated ketones with a benzophenone moiety and by simultaneously infusing the indole heterocycle, the target benzophenone-indole derivatives were obtained. The preliminary cytotoxicity screening revealed various structure–activity relationships (SARs). Tubulin polymerization inhibition evaluation, analysis of cell-cycle arrest and ROS production, MMP detection and molecular docking studies were also conducted to elucidate the derivatives’ antitumor mechanism.

## 2. Results and Discussion

### 2.1. Chemistry

**Scheme 1:**
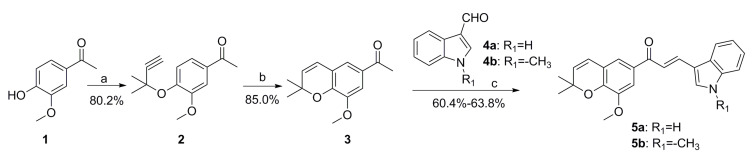
Synthesis route of target compounds **5a**-**5b**. Reagents and conditions: (**a**) 3-chloro-3-methylbut-1-yne, DBU, CuCl_2_, CH_3_CN; (**b**) pyridine, reflux; (**c**) EtOH, piperidine, reflux.

The synthesis route for target compounds **5a** and **5b** is outlined in Scheme 1. The important intermediate **3** was obtained according to the method we previously reported [21]. Then, compound **3** was reacted with different substituted indole aldehydes through aldol condensation to obtain target compound **5**.

**Scheme 2:**
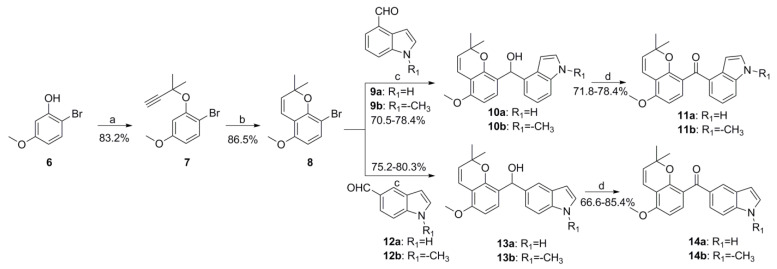
Synthesis route of target compounds **11a**-**11b** and **14a**-**14b**. Reagents and conditions: (**a**) 3-chloro-3-methylbut-1-yne, CuCl_2_, DBU, CH_3_CN, 0 °C; (**b**) pyridine, 120 °C; (**c**) n-BuLi, anhydrous THF, −78 °C; (**d**) PCC, CH_2_Cl_2_, rt.

The synthesis route of target compounds **11** and **14** is outlined in Scheme 2. Firstly, the important intermediate **8** was obtained completely according to the method we previously reported [27]. Then, compound **8** was reacted with different substituted indole aldehydes to afford diarylmethanol derivative **10** or **13** in the presence of n-BuLi. The oxidation of **10** and **13** with pyridinium chlorochromate (PCC) provided target compounds **11** and **14**, respectively.

**Scheme 3:**
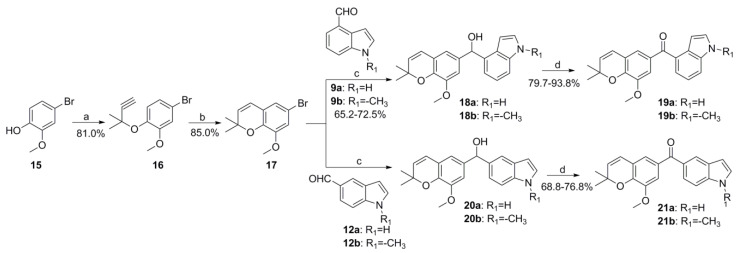
Synthesis route of target compounds **19a**-**19b** and **21a**-**21b**. Reagents and conditions: (**a**) 3-chloro-3-methylbut-1-yne, CuCl_2_, DBU, CH_3_CN, 0 °C; (**b**) pyridine,120 °C; (**c**) n-BuLi, anhydrous THF, −78 °C; (**d**) PCC, CH_2_Cl_2_, rt.

The synthesis routes of target compounds **19** and **21** in Scheme 3 are under the same procedures as described above.

### 2.2. In Vitro Antiproliferative Activity Evaluation and SAR Summary

In order to evaluate the antiproliferative activity of the designed compounds and to obtain some valuable structure–activity relationships (SARs), the initial cytotoxicity screening using a cell counting kit-8 (CCK-8) assay was performed. In the CCK-8 assay, a chemical reagent (2-(2-methoxy-4-nitrophenyl)-3-(4-nitrophenyl)-5-(2,4-disulfophenyl)-2*H*-tetrazolium sodium salt), named WST-8, which can be reduced by some dehydrogenase in mitochondria to generate orange formazan in the presence of an electron coupling reagent, was used. Hence, there is a linear relationship between the shades of orange and the number of live cells, which can be used to reflect the proliferation of cells. In our previous studies, the microtubule-targeting agents (MTAs) often displayed extraordinary cytotoxicity towards solid tumors. In this study, we also selected some common cancer cell lines, including A549 (human non-small-cell-lung cancer cell line), Caski (human epithelial cervical cancer cell line), HepG2 (human liver carcinoma cell line), C42B (Human prostate cancer line C42B) and MCF-7 (human breast carcinoma cell line) derived from different tissues for preliminary activity screening. On the base structure of millepachine, we firstly moved the position of the 2,2-dimethylbenzopyran ring of millepachine and replaced the benzene ring with the indole ring to obtain compounds **5a** and **5b**. Then, we replaced the α,β-unsaturated ketones with a benzophenone moiety to obtain compounds **11**, **14**, **19** and **21**. Overall, the effects of the position of the 2,2-dimethylbenzopyran ring, intermediate linking groups (α,β-unsaturated ketones or benzophenone moiety) and substituents on the indole ring were mainly investigated. As shown in Table 1, the general SARs were summarized as follows: (a) The reference compound millepachine displayed micromolar IC_50_ values in a range of cancer cell lines (IC_50_ = 2.566–6.712 μM); compared with millepachine, the antiproliferative activities of all of the compounds in this study have been greatly improved. (b) The introduction of the indole ring greatly improved the activity. Replacing the p-methoxyphenyl moiety of compound **A** (IC_50_ = 0.226–0.892 μM, previously reported by our group [21]) with an indole moiety to obtain the corresponding compound **14b** (IC_50_ = 0.022–0.074 μM), the antiproliferative activities were increased 27.3–78.0-fold; comparing the chemical structures of compound **14b** and compound A reminded us of the favorable effect of introducing indole moiety. (c) Moving the position of the 2,2-dimethylbenzopyran ring of millepachine was unfavorable to the activity, evidenced by comparing the activities of compounds **11a** vs. **19a**, **11b** vs. **19b**, **14a** vs. **21a** and **14b** vs. **21b**. (d) *N*-methylation of the indole ring was favorable for the activity. By comparing the antiproliferative activity results of compounds **5a** vs. **5b**, **11a** vs. **11b**, **14a** vs. **14b**, **19a** vs. **19b** and **21a** vs. **21b**, the activities were generally improved by about 1.2–4.4 folds due to *N*-methylation. (e) The position of aldol condensation has a great effect on the activity. The indole-5-carbaldehyde-derived products displayed better activities than the corresponding indole-4-carbaldehyde products, with the antiproliferative activities improved by 6.13–24.11 folds, for example, comparing the results of compounds **11a** vs. **14a**, **11b** vs. **14b**, **19a** vs. **21a** and **19b** vs. **21b**. In summary, from the rational design and activity screening, we obtained some valuable SARs and chose compound **14b** with as remarkable a cytotoxicity as the optimized compound for further study.

### 2.3. Cytotoxicity of Compound **14b** in Human Normal Cells Activities

High cytotoxicity is one of the most limiting factors in chemotherapeutics. Hence, the cytotoxicity of compound **14b** towards three kinds of human non-tumorigenic cell lines including L-02 (normal human hepatocytes), RWPE-1 (human prostate epithelial cells) and MCF-10A (normal human mammary epithelial cells) was also evaluated. As shown in Table 2, compound **14b** has much less cytotoxicity against normal cells, which was evidenced by the high selectivity ratio (16.97–231.7) between the human non-tumorigenic cell lines and the corresponding human cancer cell lines.

### 2.4. Cytotoxicity of Compound **14b** towards Drug-Resistant Cancer Cell Lines

Besides the high cytotoxicity, drug resistance is the other limiting factor of chemotherapeutics. In this study, the cytotoxicities of compound **14b** towards five kinds of drug-resistant cancer cell lines were also evaluated. Except three kinds of cell lines that are resistant to three drugs commonly used in clinical practice, two kinds of cell lines that are resistant to MTAs were also evaluated. As listed in Table 3, the resistance index of compound **14b** was 0.82–1.88, which demonstrated that **14b** exhibited almost similar potent activities between the parental cells and the drug-resistant cells.

### 2.5. Compound **14b** Inhibited Tubulin Polymerization

**Figure 2:**
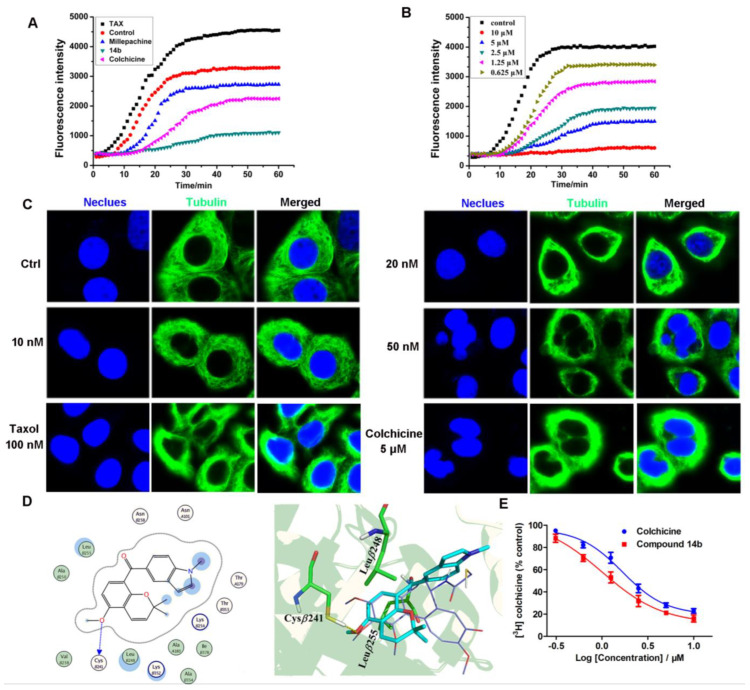
Effect of compound **14b** on tubulin. (**A**,**B**) In vitro tubulin polymerization assay of compound **14b** (5 μM), paclitaxel (TAX, 5 μM), colchicine (5 μM) and millepachine (5 μM). (**C**) Immunofluorescence assay of compound **14b** interferes with intracellular microtubules with an LSM 570 laser confocal microscope (Carl Zeiss, Jena, Germany). A549 cells were treated with or without compound **14b** (10, 20 and 50 nM), taxol (100 nM) and colchicine (5 μM) for 12 h, and followed by immunofluorescence. The experiments were performed at least three times, and representative images are shown. Scale bars are 10 μm. (**D**) Superimposition of the docked conformation of the compound **14b** on top of the X-ray structure of DAMA-colchicine (PDB code: 1SA0). The backbone of tubulin is shown in ribbon representation (α-tubulin, yellow; β-tubulin, green). (**E**) Competitive combining capacity of compound **14b** and colchicine by the colchicine competition binding assay. The treated concentrations of compound **14b** and colchicine were 10, 5, 2.5, 1.25, 0.625 and 0.3125 μM. Additionally, the IC_50_ values of compound **14b** and colchicine when inhibiting the binding of colchicine to tubulin were 1.04 ± 0.02 μM and 1.67 ± 0.05 μM, respectively. The data were shown as mean ± SE (standard error) of at least three independent experiments.

Tubulin–microtubule homeostasis is crucial for cell mitosis. In order to investigate the effect of compound **14b** on the tubulin–microtubule system, an in vitro tubulin polymerization inhibition assay, intracellular microtubule morphology detection, a molecular docking study and a colchicine competitive inhibition assay were performed. To our knowledge, the agents that interfere with tubulin are classified as microtubule stabilizing agents (MSAs) such as paclitaxel or microtubule destabilizing agents (MDAs) such as colchicine [28]. As shown in Figure 2A and 2B, compound **14b** inhibited tubulin polymerization in a colchicine-like manner, which was completely different from that of paclitaxel. Moreover, **14b** inhibited tubulin polymerization in a dose-dependent manner (IC_50_ = 2.07 ± 0.15 μM). As shown in Figure 2C, the immunofluorescence assay demonstrated that the intracellular tubulin-microtubule homeostasis was heavily disturbed by compound **14b**, evidenced by the gradually shrunken microtubule to the nucleus, and the disappearance of slim and fibrous filaments. A molecular docking study was adopted to investigate the potential mode of compound **14b** to the colchicine binding site of α,β-tubulin. As shown in Figure 2D, **14b** could occupy the colchicine binding site of tubulin in agreement with the X-ray structure complex of colchicine-α,β-tubulin (PDB code:1SA0). The 2,2-dimethylbenzopyran moiety was positioned in the binding cavity buried in the β-subunit and formed hydrophobic interactions (σ-π conjugate) with the two key amino acids of β-tubulin (Leuβ248 and Leuβ255). The indole part of **14b** increased the hydrophobic interaction with the Met259 and Thr314 residues. In order to rationalize the increase in potency, we performed the superposition of **14b** and millepachine using a docking study. As the results show in Figure S1 in the Supplementary Materials, the indole ring of **14b** formed a π-H reaction with Ala12 of β-tubulin, which was not observed in millepachine. Additionally, the benzophenone moiety of **14b** enables it to better trap the binding cavity buried in the β-subunit. These results might partially explain the increase in potency of **14b**. Finally, the results of the colchicine competitive inhibition assay (Figure 2E) show that the IC_50_ value of compound **14b** inhibiting the binding of colchicine to tubulin was 1.04 ± 0.02 μM, which was a little better than that of the reference compound colchicine (IC_50_ = 1.67 μM), further validating the fact that compound **14b** inhibited tubulin polymerization in a colchicine-like manner.

### 2.6. Compound **14b** Induced Cell Cycle Arrest at the G2/M Phase

**Figure 3:**
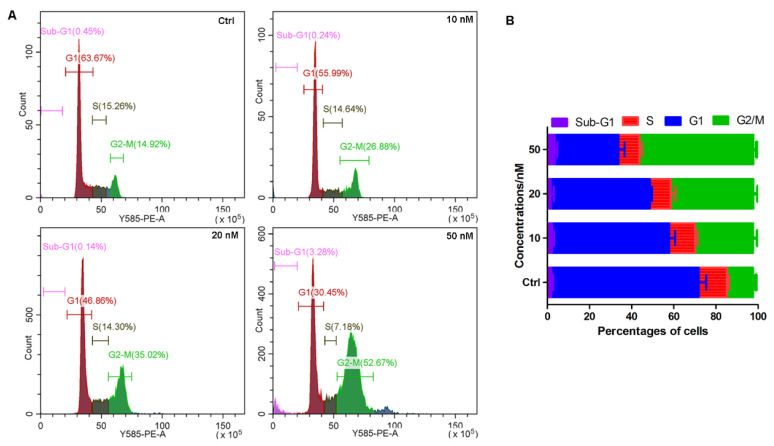
Compound **14b** induced cell cycle arrest at the G2/M phase. (**A**) The PI-stained DNA content detected by flow cytometry. A549 cells were treated with or without compound **14b** at concentrations of 10, 20 and 50 nM for 24 h; then, the cells were harvested for cell cycle analysis by flow cytometry. (**B**) Quantitative analysis of the percentage of cells in each cell cycle phase by CytExpert analysis software. The experiments were performed at least three times, and the results of the representative experiments are shown.

Microtubules play an important role in maintaining the normal process of cell mitosis. Since the above results demonstrated that compound **14b** could disturb tubulin–microtubule homeostasis, it is reasonable to speculate that compound **14b** may also interfere with the cell cycle. As the results of the flow cytometry analysis show in Figure 3, after the A549 cells were treated with compound **14b** for 24 h, the percentage of cells located in the G2/M phase increased from 14.92% in the normal group to 26.88%, 35.02% and 52.67% in the 10 nM-, 20 nM- and 50 nM-treated groups, respectively. These results demonstrated that compound **14b** induced cell cycle arrested at the G2/M phase in a dose-dependent manner.

### 2.7. Induction of Apoptosis by Compound **14b**

**Figure 4:**
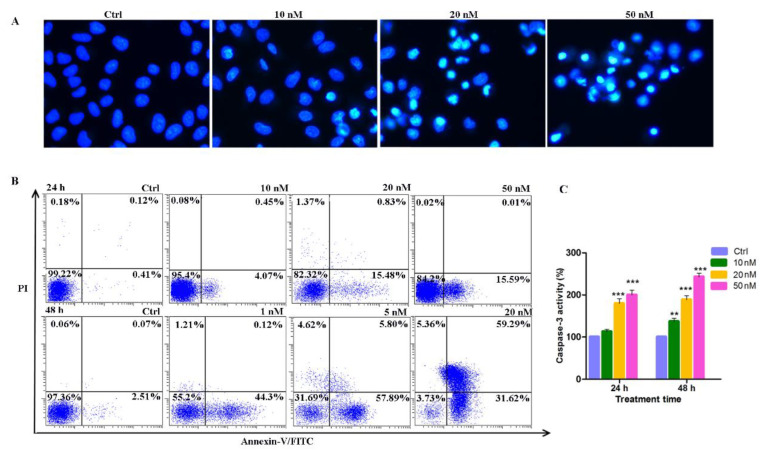
Induction of apoptosis by compound **14b**. (**A**) The Hoechst 33342 staining of A549 cells after treatment with compound **14b** at the indicated concentrations for 24 h. (**B**) The percentages of cells in each apoptosis quantitated by flow cytometry through Annexin V-FITC double staining analysis. Upper left quadrant (Annexin V^−^/PI^+^): necrotic cells; upper right quadrant (Annexin V^+^/PI^+^): late apoptotic cells; bottom left quadrant (Annexin V^−^/PI^−^): live cells; bottom right quadrant (Annexin V^+^/PI^−^): early apoptotic cells. (**C**) The caspase 3 activity detected by the caspase 3 colorimetric assay kit after A549 cells were treated with compound **14b** at concentrations of 10, 20 and 50 nM for 24 h. The experiments were performed at least three times, and the results of the representative experiments are shown. Data are presented as mean ± SEM. ** *p* < 0.01, *** *p* < 0.001 vs. control group; significant difference as compared with the control group by *t* test.

To further elucidate the mechanism of cell death caused by the cytotoxicity of compound **14b**, the Hoechst 33342 fluorochrome was used to observe the nucleus morphology, Annexin V-FITC double staining was performed to distinguish the apoptotic cells, and the caspase 3 colorimetric assay kit was used to detect the activity of caspase 3. As shown in Figure 4A, after A549 cells treated with compound **14b** at concentrations of 10, 20 and 50 nM, an increasing number of cells displayed condensed nuclei, which is a typical morphology of apoptotic cells. At the early stage of apoptosis, different types of cells turn phosphatidylserine (PS) out toward the cell surface, that is, the outside of the cell membrane, which can be selectively marked with Annexin V. Additionally, in the late stage of apoptosis, the integrity of cell membrane is lost and can be stained with PI. The flow cytometry results of Annexin V-FITC double staining in Figure 4B showed that, when A549 cells were treated with compound **14b** for 24 h, significant early apoptotic cells (Annexin V^+^/PI^−^) were observed. Additionally, when the treatment time was extended to 48 h, a gradually increasing number of late apoptotic (Annexin V^+^/PI^+^) cells were observed. These results demonstrated that the compound **14b** treatment caused cell apoptosis in a dose- and time-dependent manner. Finally, considering the activation of caspase 3 is a central event in the process of apoptosis, the activity of caspase 3 was also evaluated. As shown in Figure 4C, the activity of caspase 3 was significantly activated and increased in a dose- and time-dependent manner. In summary, the above results indicated that compound **14b** caused A549 cell death predominantly through the induction of apoptosis.

### 2.8. Compound **14b** Treatment Leads to ROS Accumulation and MMP Decrease

**Figure 5:**
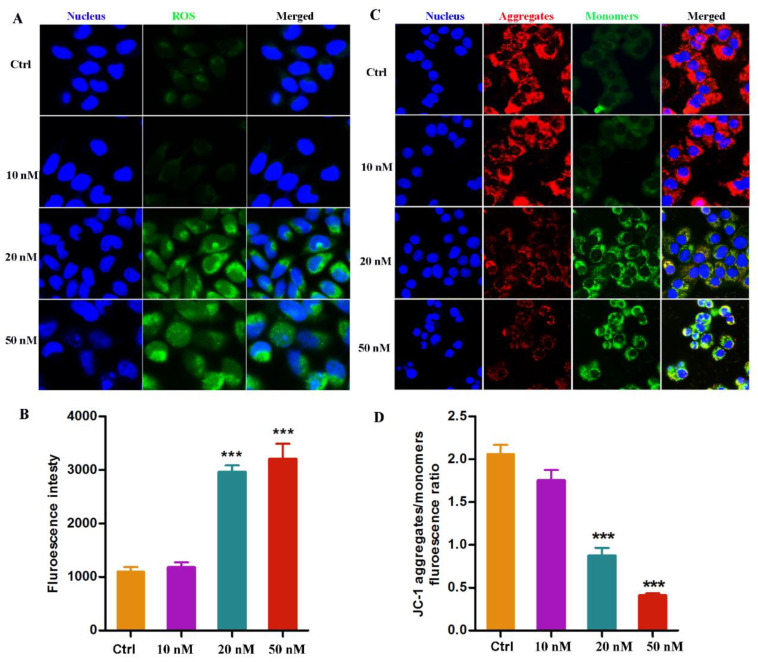
ROS accumulation and MMP alteration induced by compound **14b**. (**A**,**B**) The intracellular ROS detection by DCFH-DA after A549 cells treated with compound 14b at indicated concentrations for 12 h. (**C**,**D**) The detection of MMP monitored by JC-1 dye after A549 cells treated with compound **14b** at the concentrations of 10, 20 and 50 nM for 12 h. The experiments were performed at least three times, and the results of the representative experiments are shown. Data are presented as mean ± SEM *** *p* < 0.001 vs. Control group; significant difference as compared with the control group by *t* test. Scar bar: 10 μm.

The intrinsic mitochondrial pathway is one of the most well-studied mechanisms relevant to cell apoptosis [29]. ROS accumulation and mitochondrial membrane potential (MMP) alteration play crucial roles in this pathway. In this study, a fluorescent dye including DCFH-DA and JC-1 was used to monitor the intracellular ROS and MMP, respectively. As shown in Figure 5A, the level of ROS significantly rose abruptly in a dose-dependent manner after the A549 cells was treated with compound **14b** at concentrations of 10, 20 and 50 nM. Meanwhile, the MMP, represented by the ratio of JC-1 aggregates to monomers, also remarkably decreased, evidenced by the weakening of red fluorescence and the corresponding enhancement in green fluorescence (Figure 5B). Altogether, these results demonstrated that the treatment of compound **14b** resulted in an abnormal accumulation of ROS and a sharp decrease in MMP, which led to the dysfunction of mitochondria, thus ultimately inducing cell apoptosis.

### 2.9. The Metabolic Stability of Compound **14b**

Metabolic stability is one of the most important properties in drug development. In this study, commercially available human liver microsomes were used to evaluate the metabolic stability of compound **14b**. After incubation with human liver microsomes at 37 °C in vitro for an indicated time in a buffer solution containing a NADPH regenerating system, the residue of **14b** was monitored by HPLC. CA-4 was used as the reference compound. As shown in Table 4, compound **14b** was relatively stable in the liver microsomes. Still, 50.6% of the compound **14b** residues could be observed after 3 h of incubation; however, only 20.5% of CA-4 remained after incubation for 1 h, indicating that CA-4 displayed a relatively faster metabolic profile than **14b**. As well known, a short half-life means a low bioavailability in vivo and is required for frequent administration with a high dose, which might result in drug accumulation and may produce related toxicity. In comparison with the reference compound CA-4, which had a shorter half-life time (t_1/2_ < 60 min), the t_1/2_ of compound **14b** (t_1/2_ = 198 min) was greatly improved, which encouraged us to carry out further detailed studies in vivo.

## 3. Materials and Methods

All the chemical reagents were purchased from Aldrich (Sigma-Aldrich, St. Louis, MO, USA), Innochem. Ltd. (Shanghai, China) and Bide. Ltd. (Shanghai, China) and were used without further purification. Melting points were determined on an X-5 micromelting automated melting point apparatus. NMR spectra were recorded on a Bruker AvanceIII spectrometer with TMS as the internal standard. High-resolution mass spectra (HRMS) data were obtained by ThermoFisher TQ-S. The purity of the target compounds was determined by high-performance liquid chromatography (HPLC) with a TC-C18 column (4.6 mm × 250 mm, 5 μm) using the following conditions: injection volume: 10 μL; flow rate: 1 mL/min; and mobile phase: acetonitrile/water or methanol/water.

The cell lines used in this study were obtained from National Collection of Authenticated Cell Cultures (Shanghai, China). The tubulin polymerization kit was obtained from cytoskeleton, Inc. (cat.#BK011P, Denver, MA, USA). The CCK-8 assay kit, cell cycle analysis kit and Annexin V-FITC double staining kit were purchased from Beyotime Biotechnology (Shanghai, China). The fluorescent dye including Hoechst 33342, DCFH-DA and JC-1 were obtained from Sigma-Aldrich (St. Louis, MO, USA).

### 3.1. Chemistry

#### 3.1.1. General Procedures for Known Compounds

The preparation of the known compound **3**, compound **8** and compound **17** were carried out in complete accordance with our previously reported methods [24,26].

#### 3.1.2. General Procedures for Preparation of Compounds **5a**-**5b**

The preparation of compounds **5a**-**5c** was carried out according to our previously reported method [24]. Briefly, compound **3** (1 mmol, 232 mg) was dissolved in EtOH (4 mL), and piperidine (1.2 mmol, 0.12 mL) and indolal **4a** (0.8 mmol, 116 mg) were added in sequence to the solution. After the mixture was stirred at 95 °C for 20 h, the diluted hydrochloric acid was added to quench the reaction and to adjust the reaction system to pH = 6. The mixture was extracted with EtOAc, and the organic layer was subsequently washed with aqueous NaHCO_3_, water and brine. After drying over anhydrous Na_2_SO_4_, the mixture was concentrated under reduced pressure. The residue was purified by column chromatography with petroleum/ethyl acetate (10:1) as an eluent to afford the target compound **5a**.

(*E*)-3-(1*H*-indol-3-yl)-1-(8-methoxy-2,2-dimethyl-2*H*-chromen-6-yl)prop-2-en-1-one (**5a**)

Light yellow solid, yield: 41.2%. ^1^H NMR (400 MHz, CDCl_3_) δ 8.29 (s, 1H), 8.04 (d, *J* = 7.2 Hz, 1H), 7.92 (s, 1H), 7.87 (d, *J* = 9.8 Hz, 1H), 7.51 (s, 1H), 7.36 (d, *J* = 7.2 Hz, 1H), 7.32 (m, 2H), 7.17 (d, *J* = 8.3 Hz, 1H), 6.69 (s, 1H), 6.48 (d, *J* = 9.8 Hz, 1H), 5.18 (d, *J* = 9.8 Hz, 1H), 3.90 (s, 3H), 1.62 (s, 6H). ^13^C NMR (101 MHz, CDCl_3_) δ 191.11 (CO, C-7), 146.35 (C, C-3), 145.42 (C, C-2), 139.07 (C, C-18), 134.22 (C, C-5), 132.41 (CH, C-9), 131.45 (CH, C-11), 130.31 (CH, C-17), 126.28 (C, C-19), 123.95 (C, C-6), 123.43 (CH, C-8), 122.45 (CH, C-10), 122.33 (CH, C-21), 120.92 (C, C-1), 120.62 (CH, C-23), 120.17 (CH, C-22), 116.71 (C, C-16), 112.77 (CH, C-20), 110.58 (CH, C-4), 77.53 (C, C-12), 56.28 (CH_3_, C-15), 28.04 (CH_3_, C-13, 14). HRMS (ESI) (m/z) [M+H]^+^calcd for C_23_H_22_NO_3_, 360.15942; found, 360.15952. Purity: 97.7% (by HPLC).

(*E*)-1-(8-methoxy-2,2-dimethyl-2*H*-chromen-6-yl)-3-(1-methyl-1*H*-indol-3-yl)prop-2-en-1-one (**5b**)

Light yellow solid, yield: 43.5%. ^1^H NMR (400 MHz, CDCl_3_) δ 8.14 (d, *J* = 16.0 Hz, 1H), 7.80 (d, *J* = 9.8 Hz, 1H), 7.66 (d, *J* = 8.7 Hz, 1H), 7.55 (s, 1H), 7.46 (s, 1H), 7.13 (d, *J* = 8.7 Hz, 1H), 7.00 (d, *J* = 8.1 Hz, 1H), 6.76 (d, *J* = 8.8 Hz, 1H), 6.61 (d, *J* = 7.8 Hz, 1H), 6.54 (d, *J* = 8.8 Hz, 1H), 5.18 (d, *J* = 9.8 Hz, 1H), 3.92 (s, 3H), 3.89 (s, 3H), 1.62 (s, 6H). ^13^C NMR (101 MHz, CDCl_3_) δ 190.21 (CO, C-7), 146.35 (C, C-3), 145.42 (C, C-2), 138.52 (C, C-18), 136.98 (C, C-5), 134.22 (CH, C-9), 132.99 (CH, C-11), 130.51 (CH, C-17), 125.01 (C, C-19), 124.49 (C, C-6), 123.85 (CH, C-8), 122.65 (CH, C-10), 122.35 (CH, C-21), 120.58 (C, C-1), 120.36 (CH, C-23), 118.83 (CH, C-22), 113.41 (C, C-16), 112.87 (CH, C-20), 110.29 (CH, C-4), 76.52 (C, C-12), 56.28 (CH_3_, C-15), 33.41 (CH_3_, C-24), 28.27 (CH_3_, C-13, 14). HRMS (ESI) (m/z) [M+H]^+^ calcd for C_24_H_24_NO_3_, 374.17507; found, 374.17511 Purity: 99.2% (by HPLC).

#### 3.1.3. General Procedures for Preparation of Compounds **11a-11b**, **14a-14b**, **19a-19b** and **21a-21b**

The preparation of compounds **11a-11b**, **14a-14b**, **19a-19b** and **21a-21b** was carried out according to our previously reported method [27,30]. To a solution of compound **8** (1.0 mmol, 269 mg) in 20 mL of anhydrous THF in nitrogen atmosphere at −78 °C, n-butyllithium (0.8 mL, 2.5 M in hexane) was added in dropwise. After the solution was stirred for 0.5 h, commercially available compound **9a** (1.0 mmol, 145 mg) was added, and then, the reaction mixture was stirred overnight in darkness. A saturated NH_4_Cl solution was added, and the mixture was extracted with EtOAc, and the organic layer was subsequently washed with aqueous NaHCO_3_, water and brine. After drying over anhydrous Na_2_SO_4_, the mixture was concentrated under reduced pressure to obtain the crude product **10a**, which was used directly in the next step of the reaction without further purification.

Compound **10a** (1 mmol, 335 mg) was dissolved in dichloromethane; then, PCC (1.2 mmol, 451 mg) and silica gel (451 mg) were added to the mixture and stirred at room temperature for 5–8 h. After the reaction was completed, the suspension was filtered and the solvent was concentrated under a vacuum to provide the crude product, which was subsequently purified by silica gel column chromatography with petroleum/ethyl acetate (10:1–3:1) as an eluent to afford the target compound **11a**.

(1*H*-indol-4-yl)(5-methoxy-2,2-dimethyl-2*H*-chromen-8-yl)methanone (**11a**)

White solid, yield: 39.8%. ^1^H NMR (400 MHz, CDCl_3_) δ 8.18 (s, 1H), 7.50 (d, *J* = 9.8 Hz, 1H), 7.46 (d, *J* = 16.0 Hz, 1H), 7.21 (d, *J* = 7.8 Hz, 1H), 7.17 (d, *J* = 7.8 Hz, 1H), 7.01 (d, *J* = 8.7 Hz, 1H), 6.84 (d, *J* = 11 Hz, 1H), 6.67 (d, *J* = 7.8 Hz, 1H), 6.59 (d, *J* = 7.8 Hz, 1H), 5.46 (d, *J* = 9.8 Hz, 1H), 3.90 (s, 3H), 1.53 (s, 6H). ^13^C NMR (101 MHz, CDCl_3_) δ 191.91 (CO, C-7), 155.26 (C, C-2), 152.41 (C, C-6), 135.06 (C, C-12), 130.57 (C, C-13), 127.98 (CH, C-15), 127.56 (CH, C-9), 127.06 (C, C-8), 126.64 (CH, C-4), 125.84 (CH, C-21), 124.81 (CH, C-10), 117.53 (CH, C-16), 116.31 (C, C-5), 107.82 (CH, C-3), 106.72 (CH, C-11), 104.90 (C, C-1), 101.44 (CH, C-20), 76.70 (C, C-14), 55.35 (CH_3_, C-17), 27.85 (CH_3_, C-18,C-19). HRMS (ESI) (m/z) [M+H]^+^calcd for C_21_H_20_NO_3_, 334.14377; found, 334.14430. Purity: 98.9% (by HPLC).

(5-methoxy-2,2-dimethyl-2H-chromen-8-yl)(1-methyl-1H-indol-4-yl)methanone (**11b**)

White solid, yield: 40.5%. ^1^H NMR (400 MHz, CDCl_3_) δ 7.56 (d, *J* = 4.8 Hz, 1H), 7.46 (d, *J* = 11.0 Hz, 1H), 7.17 (d, *J* = 7.8 Hz, 1H), 7.14 (d, *J* = 7.8 Hz, 1H), 6.95 (d, *J* = 9.8 Hz, 1H), 6.80 (d, *J* = 11 Hz, 1H), 6.64 (d, *J* = 9.8 Hz, 1H), 6.55 (d, *J* = 7.8 Hz, 1H), 5.42 (d, *J* = 9.8 Hz, 1H), 3.91 (s, 3H), 3.85 (s, 3H),1.51 (s, 6H). ^13^C NMR (101 MHz, CDCl_3_) δ 195.21 (CO, C-7), 157.58 (C, C-2), 155.45 (C, C-6), 136.55 (C, C-12), 131.47 (C, C-13), 130.48 (CH, C-15), 129.13 (CH, C-9), 128.33 (C, C-8), 127.03 (CH, C-4), 126.10 (CH, C-21), 123.50 (CH, C-10), 117.66 (CH, C-16), 116.58 (C, C-5), 113.42 (CH, C-3), 112.26 (CH, C-11), 107.61 (C, C-1), 100.57 (CH, C-20), 76.48 (C, C-14), 56.01 (CH_3_, C-17), 32.55 (CH_3_, C-22), 27.87 (CH_3_, C-18,19). HRMS (ESI) (m/z) [M+H]^+^calcd for C_22_H_22_NO_3_, 348.15942; found, 348.15968. Purity: 99.2% (by HPLC).

(1H-indol-5-yl)(5-methoxy-2,2-dimethyl-2H-chromen-8-yl)methanone (**14a**)

White solid, yield: 46.2%. ^1^H NMR (400 MHz, CDCl_3_) δ 8.66 (s, 1H), 8.32 (s, 1H), 7.63 (d, *J* = 7.8 Hz, 1H), 7.53 (d, *J* = 9.8 Hz, 1H), 7.49 (d, *J* = 7.8 Hz, 1H), 7.39 (d, *J* = 7.8 Hz, 1H), 6.52 (d, *J* = 79.8 Hz, 1H), 6.45-6.48 (m, 2H), 5.23 (d, *J* = 9.8 Hz, 1H), 3.85 (s, 3H), 1.52 (s, 6H). ^13^C NMR (101 MHz, CDCl_3_) δ 195.58 (CO, C-7), 157.42 (C, C-2), 155.12 (C, C-6), 138.41 (C, C-11), 129.16 (C, C-8), 127.82 (CH, C-12), 127.13 (CH, C-17), 125.80 (CH, C-4), 125.42 (CH, C-14), 125.18 (CH, C-9), 121.50 (CH, C-13), 117.62 (CH, C-18), 117.41 (C, C-5), 111.27 (CH, C-3), 109.37 (CH, C-10), 106.95 (C, C-1), 102.81 (CH, C-15), 77.68 (C, C-16), 55.91 (CH_3_, C-19), 27.82 (CH_3_, C-20, 21). HRMS (ESI) (m/z) [M+H]^+^ calcd for C_21_H_20_NO_3_, 334.14377; found, 334.14382. Purity: 98.7% (by HPLC).

(5-methoxy-2,2-dimethyl-2H-chromen-8-yl)(1-methyl-1H-indol-5-yl)methanone (**14b**)

White solid, yield: 38.5%. ^1^H NMR (400 MHz, CDCl_3_) δ 8.35 (s, 1H), 7.78 (d, *J* = 7.8 Hz, 1H), 7.63 (d, *J* = 9.8 Hz, 1H), 7.51 (d, *J* = 7.8 Hz, 1H), 7.11 (d, *J* = 7.8 Hz, 1H), 6.62 (d, *J* = 9.8 Hz, 1H), 6.57–6.55 (m, 2H), 5.28 (d, *J* = 9.8 Hz, 1H), 3.90 (s, 3H), 3.78 (s, 3H), 1.54 (s, 6H). ^13^C NMR (101 MHz, CDCl_3_) δ 195.47 (CO, C-7), 157.50 (C, C-2), 155.22 (C, C-6), 139.27 (C, C-11), 130.17 (C, C-8), 129.02 (CH, C-12), 128.18 (CH, C-17), 127.03 (CH, C-4), 125.80 (CH, C-14), 125.14 (CH, C-9), 122.24 (CH, C-13), 117.56 (CH, C-18), 117.40 (C, C-5), 111.97 (CH, C-3), 109.11 (CH, C-10), 107.19 (C, C-1), 102.88 (CH, C-15), 77.88 (C, C-16), 56.26 (CH_3_, C-19), 32.81 (CH_3_, C-22), 27.82 (CH_3_, C-20, 21). HRMS (ESI) (m/z) [M+H]^+^calcd for C_22_H_22_NO_3_, 348.15942; found, 348.15921. Purity: 96.6% (by HPLC).

(1H-indol-4-yl)(8-methoxy-2,2-dimethyl-2H-chromen-6-yl)methanone (**19a**)

Light yellow solid, yield:42.1%. ^1^H NMR (400 MHz, CDCl_3_) δ 8.10 (s, 1H), 7.81 (d, *J* = 9.8 Hz, 1H), 7.67 (d, *J* = 9.8 Hz, 1H), 7.50 (s, 1H), 7.39 (t, *J* = 7.8 Hz, 1H), 7.28 (d, *J* = 7.8 Hz, 1H), 7.19 (s, 1H), 6.43 (d, *J* = 7.8 Hz, 1H), 6.37 (d, *J* = 7.8 Hz, 1H), 5.23 (d, *J* = 11.0 Hz, 1H), 3.90 (s, 3H), 1.61 (s, 6H). ^13^C NMR (101 MHz, CDCl_3_) δ 195.10 (CO, C-7), 145.98 (C, C-3), 144.75 (C, C-2), 135.97 (C, C-12), 133.80 (C, C-5), 131.51 (CH, C-15), 130.07 (C, C-13), 127.80 (C, C-8), 126.93 (CH, C-9), 124.94 (CH, C-20), 123.12 (CH, C-10), 122.76 (CH, C-6), 121.98 (CH, C-14), 120.05 (C, C-1), 111.64 (CH, C-11), 108.89 (CH, C-4), 101.31 (CH, C-21), 77.82 (C, C-16), 55.68 (CH_3_, C-19), 27.97 (CH_3_, C-17,18). HRMS (ESI) (m/z) [M+H]^+^ calcd for C_21_H_20_NO_3_, 334.14377; found, 334.14310. Purity: 98.2% (by HPLC).

(8-methoxy-2,2-dimethyl-2H-chromen-6-yl)(1-methyl-1H-indol-4-yl)methanone (**19b**)

Light yellow solid, yield:39.8%. ^1^H NMR (400 MHz, CDCl_3_) δ7.74 (d, *J* = 8.8 Hz, 1H), 7.63 (d, *J* = 8.8 Hz, 1H), 7.51 (s, 1H), 7.38 (t, *J* = 7.8 Hz, 1H), 7.31 (d, *J* = 7.8 Hz, 1H), 7.21 (s, 1H), 6.49 (d, *J* = 11.0 Hz, 1H), 6.43 (d, *J* = 7.8 Hz, 1H), 5.23 (d, *J* = 11.0 Hz, 1H), 3.86 (s, 3H), 3.78 (s, 3H), 1.62 (s, 6H). ^13^C NMR (101 MHz, CDCl_3_) δ 195.89 (CO, C-7), 146.28 (C, C-3), 144.75 (C, C-2), 137.06 (C, C-12), 134.63 (C, C-5), 132.81 (CH, C-15), 131.83 (C, C-13), 131.15 (C, C-8), 130.14 (CH, C-9), 129.13 (CH, C-20), 123.44 (CH, C-10), 122.88 (CH, C-6), 122.35 (CH, C-14), 120.26 (C, C-1), 111.44 (CH, C-11), 110.58 (CH, C-4), 100.57 (CH, C-21), 77.88 (C, C-16), 56.28 (CH_3_, C-19), 33.53 (CH_3_, C-22), 28.33 (CH_3_, C-17,18). HRMS (ESI) (m/z) [M+H]^+^calcd for C_22_H_22_NO_3_, 348.15942; found, 348.15968. Purity: 97.9% (by HPLC).

(1H-indol-5-yl)(8-methoxy-2,2-dimethyl-2H-chromen-6-yl)methanone (**21a**)

Light yellow solid, yield:36.2%. ^1^H NMR (400 MHz, CDCl_3_) δ 8.68 (s, 1H), 8.28 (s, 1H), 7.76 (d, *J* = 8.8 Hz, 1H), 7.57 (d, *J* = 7.8 Hz, 1H), 7.51 (s, 1H), 7.29 (d, *J* = 7.8 Hz, 1H), 7.15 (s, 1H), 6.63 (d, *J* = 8.8 Hz, 1H), 6.47 (d, *J* = 11.0 Hz, 1H), 5.19 (d, *J* = 10.8 Hz, 1H), 3.89 (s, 3H), 1.61 (s, 6H). ^13^C NMR (101 MHz, CDCl_3_) δ 194.92 (CO, C-7), 146.09 (C, C-3), 144.62 (C, C-2), 138.40 (C, C-11), 133.64 (C, C-5), 131.41 (CH, C-17), 129.03 (C, C-8), 127.19 (C, C-12), 125.80 (CH, C-9), 125.39 (CH, C-14), 123.22 (CH, C-6), 122.35 (CH, C-16), 121.11 (CH, C-13), 120.11 (C, C-1), 109.48 (CH, C-4), 109.36 (CH, C-10), 102.71 (CH, C-15), 77.93 (C, C-18), 56.12 (CH_3_, C-21), 28.05 (CH_3_, C-19, 20). HRMS (ESI) (m/z) [M+H]^+^calcd for C_21_H_20_NO_3_, 334.14377; found, 334.14570. Purity: 98.0% (by HPLC).

(8-methoxy-2,2-dimethyl-2H-chromen-6-yl)(1-methyl-1H-indol-5-yl)methanone (**21b**)

Light yellow solid, yield:39.1%. ^1^H NMR (400 MHz, CDCl_3_) δ 8.41 (s, 1H), 7.79 (d, *J* = 7.8 Hz, 1H), 7.69 (d, *J* = 8.8 Hz, 1H), 7.48 (s, 1H), 7.23 (s, 1H), 7.19 (d, *J* = 7.8 Hz, 1H), 6.70 (d, *J* = 8.8 Hz, 1H), 6.52 (d, *J* = 11.0 Hz, 1H), 5.19 (d, *J* = 11.0 Hz, 1H), 3.90 (s, 3H), 3.80 (s, 3H), 1.62 (s, 6H). ^13^C NMR (101 MHz, CDCl_3_) δ 195.12 (CO, C-7), 146.22 (C, C-3), 145.02 (C, C-2), 139.82 (C, C-11), 133.94 (C, C-5), 131.71 (CH, C-17), 128.82 (C, C-8), 128.61 (C, C-12), 127.36 (CH, C-9), 125.15 (CH, C-14), 123.42 (CH, C-6), 122.65 (CH, C-16), 121.73 (CH, C-13), 119.91 (C, C-1), 109.72 (CH, C-4), 108.49 (CH, C-10), 102.68 (CH, C-15), 77.89 (C, C-18), 56.38 (CH_3_, C-21), 32.67 (CH_3_, C-22), 28.07 (CH_3_, C-19, 20). HRMS (ESI) (m/z) [M+H]^+^calcd for C_22_H_22_NO_3_, 348.15942; found, 348.16061. Purity: 98.2% (by HPLC).

### 3.2. Cell Lines and Cell Culture

A549 (non-small-cell-lung cancer cell line), Caski (human epithelial cervical cancer cell line), HepG2 (human liver carcinoma cell line), MCF-7 (human breast carcinoma cell line), C42B (human prostate cancer line C42B), L-02 (human normal hepatocytes), RWPE-1 (human prostate epithelial cells) and MCF-10A (human normal mammary epithelial cells) were obtained from National Collection of Authenticated Cell Cultures (Shanghai, China). A549/CDDP (A549 cell line resistant to cisplatin), C42B/ENZR (C42b cell line resistant to Enzalutamide), MCF-7/DOX (MCF-7 cell line resistant to doxorubicin), A2780/TAX (human ovarian cancer cell line A2780 resistant to taxol) and HCT-8/VCR (human ileocecum carcinoma cell lines HCT-8 resistant to vincristine) were kindly provided by Professor Junjian Wang and Professor Xingshu Li at Sun Yat-Sen University, China. All cell lines were cultivated in Dulbecco’s modified Eagle’s medium (DMEM) or RPMI-1640 medium containing 10% (*v*/*v*) heat-inactivated FBS, 100 units/mL penicillin and 100 mg/mL streptomycin and cultured in a humidified atmosphere of 5% CO_2_ at 37 °C.

### 3.3. Biological Evaluation Assays

The biological evaluation applied in this study, including a CCK-8 assay, an in vitro tubulin polymerization inhibition assay, a molecular docking study, colchicine competitive inhibition assay intracellular microtubule morphology detection using immunofluorescence, a cell cycle and apoptosis assay using flow cytometry, a caspase 3 activity evaluation, and intracellular ROS and MMP detection were performed completely under the guidance of our previously published protocols [24,25,26]. These protocols are briefly described in the Supplementary Materials.

## 4. Conclusions

Natural-product-based drug discovery provides a strong basis for the development of antitumor drugs. In this study, under the guidance of our previously obtained valuable SARs and starting from the structure of millepachine, a natural product from the seeds of Millettia pachycarpa, a rational drug design strategy that included replacing the α,β-unsaturated ketones with a benzophenone moiety and simultaneously infusing the indole heterocycle was applied. The optimized compound **14b** displayed excellent antiproliferative activities towards a panel of human cancer cell lines (0.022–0.074 μM), which was almost 100-fold more active than millepachine (IC_50_ = 2.566–6.712 μM). For target exploration, some experiments used a tubulin–microtubule system to validate that compound **14b** inhibited tubulin polymerization. As with most microtubule target agents, compound **14b** inhibited tubulin polymerization by binding to the colchicine binding site of tubulin; disturbed the equilibrium of the tubulin–microtubule system; arrested the cell cycle at the G2/M phase; and therefore, interfered with mitosis. The **14b**-induced apoptosis was accompanied by the activation of caspase 3, an abnormal accumulation of ROS and a decrease in MMP, which ultimately led to cancer cell death. Meanwhile, **14b** displayed nearly equally potent antiproliferative activity toward drug-resistant cells and parent cancer cells, which provides a potential for **14b** co-administration with currently clinically used anticancer drugs. Finally, the extraordinary in vitro anticancer activity and metabolic stability in human liver microsomes of compound **14b** encourages us to evaluate its in vivo antitumor activity in a further study. Altogether, these results demonstrate the promising worth of **14b** as a potential chemotherapeutic agent.

## Figures and Tables

**Table 1:** The antiproliferative activities evaluated in five kinds of human cancer cell lines ^a^.

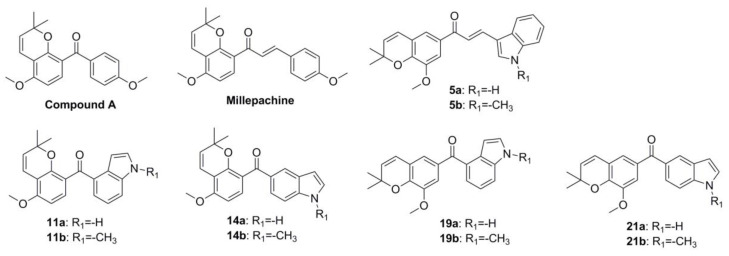					
Compds	IC_50_, Mean ± SE (μΜ) ^b^				
	A549	Caski	HepG2	C42B	MCF-7
5a	0.812 ± 0.022	1.929 ± 0.035	0.963 ± 0.032	1.377 ± 0.021	1.553 ± 0.014
5b	0.362 ± 0.017	0.729 ± 0.033	0.461 ± 0.017	0.577 ± 0.021	0.350 ± 0.011
11a	0.631 ± 0.018	0.832 ± 0.011	0.998 ± 0.034	0.911 ± 0.034	0.816 ± 0.045
11b	0.208 ± 0.009	0.516 ± 0.012	0.454 ± 0.021	0.455 ± 0.015	0.651 ± 0.024
14a	0.072 ± 0.004	0.086 ± 0.009	0.142 ± 0.014	0.089 ± 0.021	0.082 ± 0.011
14b	0.022 ± 0.002	0.037 ± 0.003	0.074 ± 0.013	0.051 ± 0.005	0.027 ± 0.006
19a	1.321 ± 0.016	2.311 ± 0.019	3.098 ± 0.021	2.411 ± 0.042	1.775 ± 0.004
19b	0.642 ± 0.081	1.098 ± 0.032	0.898 ± 0.009	1.076 ± 0.034	1.021 ± 0.015
21a	0.105 ± 0.011	0.211 ± 0.012	0.441 ± 0.071	0.209 ± 0.020	0.121 ± 0.021
21b	0.082 ± 0.010	0.106 ± 0.012	0.097 ± 0.031	0.107 ± 0.006	0.099 ± 0.031
Compd. A	0.226 ± 0.035	0.892 ± 0.024	0.758 ± 0.017	0.526 ± 0.007	0.456 ± 0.021
Millepachine	2.566 ± 0.131	6.712 ± 0.233	3.881 ± 0.244	3.176 ± 0.384	4.528 ± 0.338

^a^ Cell lines were treated with different concentrations of the compounds for 48 h. Cell viability was measured by a CCK-8 assay, as described in the Materials and Methods section. ^b^ IC_50_ values are indicated as the mean ± SE (standard error) of at least three independent experiments.

**Table 2:** Cytotoxicity selectivity of compound **14b** toward human non-tumorigenic cell lines and cancer cell lines ^a^.

Cell Lines	IC_50_, Mean ± SE (μM) ^b^	Selectivity Ratio ^c^
Hep-G2	0.074 ± 0.013	16.97
L-02	1.256 ± 0.214	
C42B	0.051 ± 0.005	72.51
RWPE-1	3.698 ± 0.036	
MCF-7	0.027 ± 0.006	231.7
MCF-10A	6.256 ± 0.311	

^a^ Cell lines were treated with different concentrations of the compounds for 48 h. Cell viability was measured by a CCK-8 assay, as described in the Materials and Methods section. ^b^ IC_50_ values are indicated as the mean ± SE (standard error) of at least three independent experiments. ^c^ Selectivity ratio = (IC_50_ of human normal cells)/(IC_50_ of corresponding cancer cell lines).

**Table 3:** Cytotoxicity of compound **14b** toward drug-resistant cancer cell lines ^a^.

Cell Lines	IC_50_, Mean ± SE (μM) ^b^	Resistance Index ^c^
A549	0.022 ± 0.002	1.59
A549/CDDP ^d^	0.035 ± 0.021	
C42B	0.051 ± 0.005	0.82
C42B/ENZR ^e^	0.042 ± 0.011	
MCF-7	0.027 ± 0.006	1.88
MCF-7/DOX ^f^	0.051 ± 0.024	
A2780	0.036 ± 0.004	1.17
A2780/TAX ^g^	0.042 ± 0.011	
HCT-8	0.089 ± 0.015	1.37
HCT-8/VCR ^h^	0.122 ± 0.036	

^a^ Cell lines were treated with different concentrations of the compounds for 48 h. Cell viability was measured by a CCK-8 assay, as described in the Materials and Methods section. ^b^ IC_50_ values are indicated as the mean ± SE (standard error) of at least three independent experiments. ^c^ Resistance index = (IC_50_ of drug-resistant cell lines)/(IC_50_ of corresponding parent cancer cell lines); ^d^ A549/CDDP: A549 cell line resistant to cisplatin; ^e^ C42B/ENZR: C42b cell line resistant to Enzalutamide; ^f^ MCF-7/DOX: MCF-7 cell line resistant to doxorubicin; ^g^ A2780/TAX: A2780 cell line resistant to taxol; ^h^ HCT-8/VCR: HCT-8 cell line resistant to vincristine.

**Table 4:** The metabolic stability of **14b** and CA-4.

Compd.	Time/min, Remaining%							t_1/2_, min
	30	60	90	120	150	180	240	
**14b**	96.18%	91.38%	84.14%	74.45%	67.75%	56.17%	43.43%	198
CA-4	70.2%	28.3%	20.5%	18.7%	NT ^a^	NT	NT	ND ^b^

^a^ NT: not tested because of the fast metabolic rate of CA-4; ^b^ ND: not determined.

## Data Availability

The data used in this study are presented in this article.

## Supplementary Materials

The following supporting information can be downloaded at https://www.mdpi.com/article/10.3390/molecules28031481/s1, S I: Figure S1. The superposition of 14b and millepachine using docking study; S II: NMR spectra of target compounds (S2–S11); S III: HPLC chromatograms of target compounds (S12–S15); S IV: High resolution mass spectra of target compounds (S16–S25); S V: Biological evaluation methods (S26–S27).

## Sample Availability

Samples of the compounds are available from the authors.
