# Supplementary material for: Novel Indole-Containing Hybrids Derived from Millepachine: Synthesis, Biological Evaluation and Antitumor Mechanism Study
Source: Molecules. 2023 Feb 3;28(3):1481. doi: 10.3390/molecules28031481 (PMC9921564; doi:10.3390/molecules28031481)
Supplement: Supplementary file 1 [file molecules-28-01481-s001.zip › molecules-2127417-supplementary.pdf]

Supporting Information

Novel Indole-Containing Hybrids Derived from Millepachine: Synthesis, Biological Evaluation and Antitumor Mechanism Study

Baoxia Liang [1,*], Qing Zou [1], Lintao Yu [2], Yali Wang [3], Jun Yan [2] and Baiqi Huang [1]

[1] The School of Food Science and Biology, Guangdong Polytechnic of Science and Trade, Guangzhou 510430, China
[2] Second Clinical Medical College, Guangzhou University of Chinese Medicine, Guangzhou 510120, China
[3] BGI Infection Pharmaceutical Technology, BGI-Shenzhen, Shenzhen 518083, China
* Correspondence: liangbaoxia@gzhmu.edu.cn

S I. NMR spectra of target compounds（S2-S11）

S II. HPLC chromatograms of target compounds (S12 – S15)

S Ⅲ. High resolution mass spectra of target compounds（S16 – S25）

S Ⅳ. Biological evaluation methods （S26 – S27）

S I. NMR spectrums of target compounds

5a

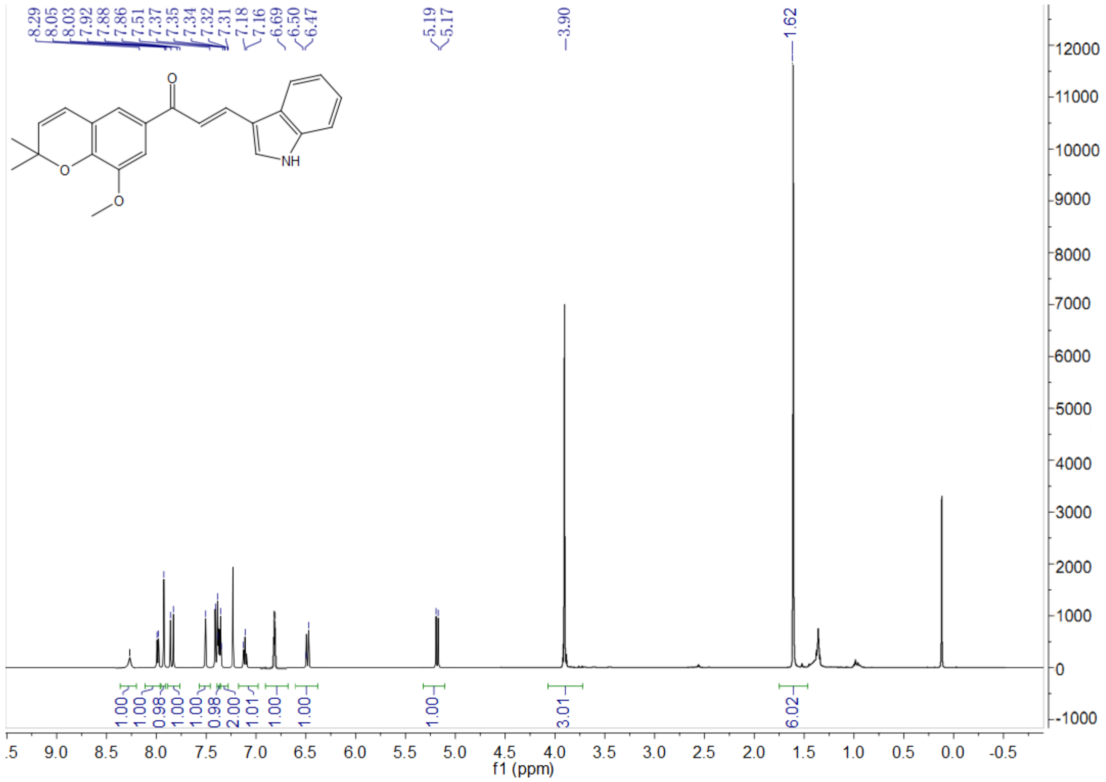
8.29
8.05
8.03
7.92
7.88
7.86
7.51
7.37
7.35
7.34
7.32
7.31
7.18
7.16
6.69
6.50
6.47
5.19
5.17
3.90
1.62
O
NH
O
O
1.00
1.00
0.98
1.00
1.00
0.98
2.00
1.01
1.00
1.00
1.00
3.01
6.02
f1 (ppm)

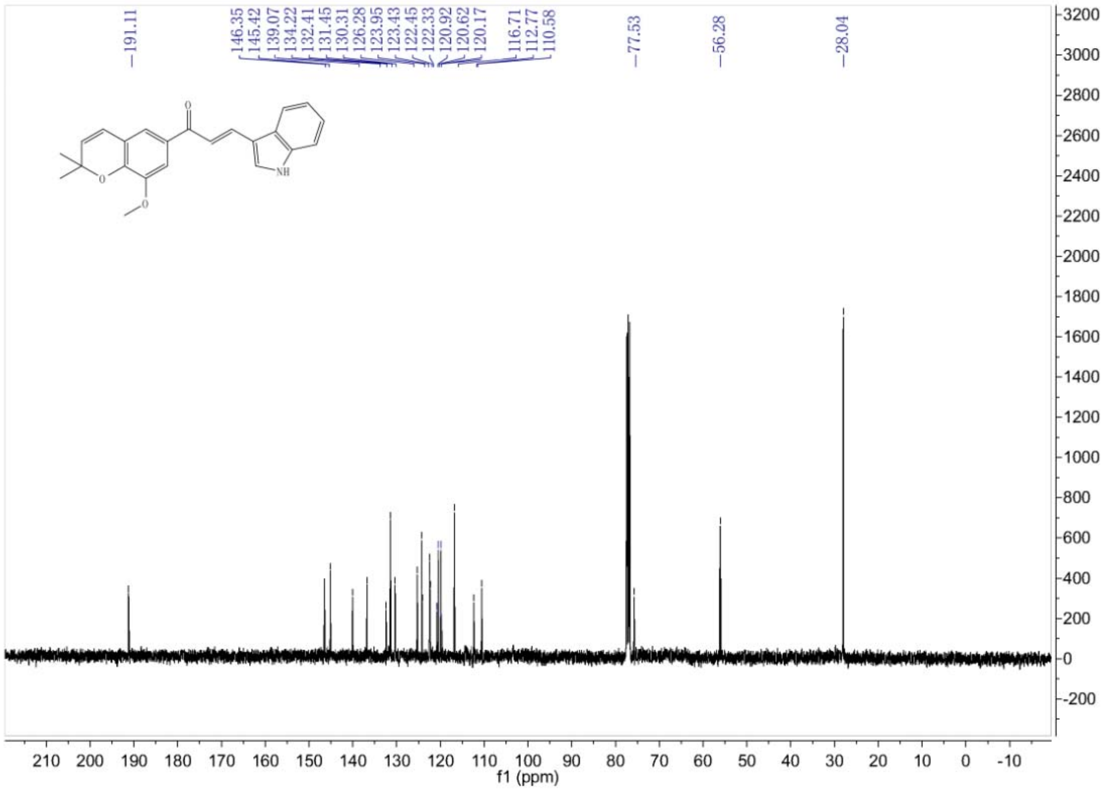
191.11
146.35
145.42
139.07
134.22
132.41
131.45
130.31
126.28
123.95
123.43
122.45
122.33
120.92
120.62
120.17
116.71
112.77
110.58
77.53
56.28
28.04
O
NH
O
O
f1 (ppm)

5b

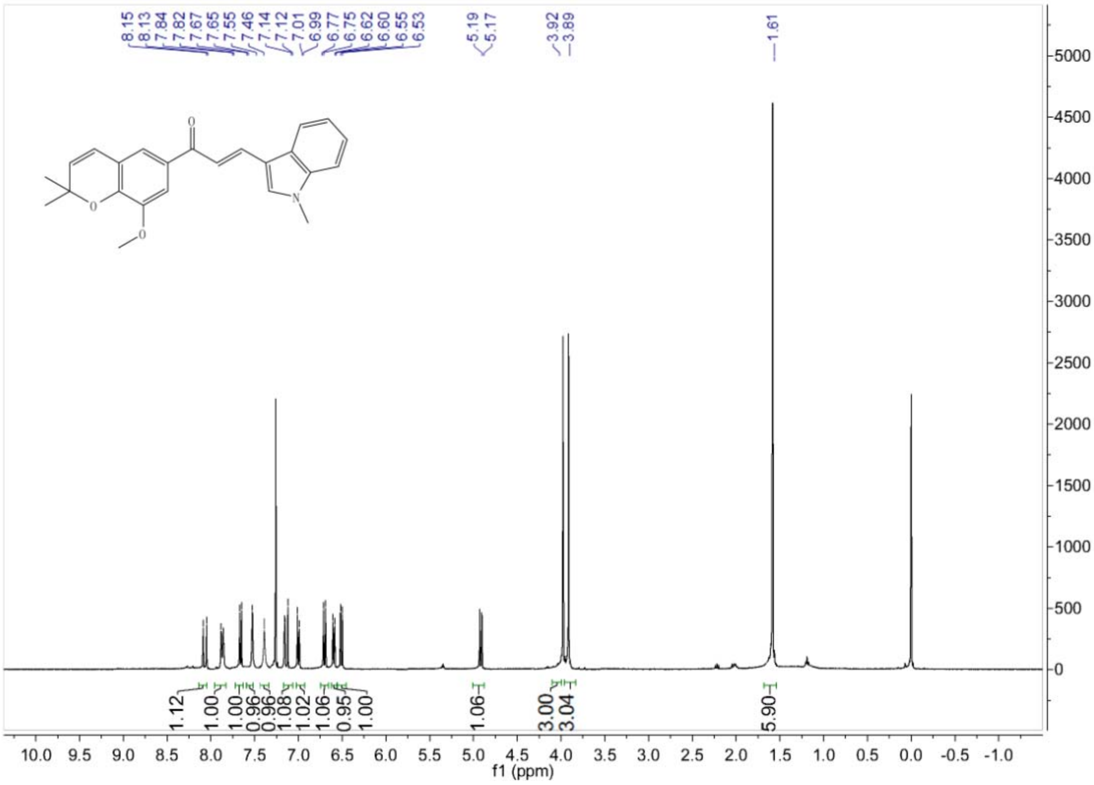
8.15 8.13 7.84 7.82 7.67 7.65 7.55 7.46 7.14 7.12 7.01 6.99 6.77 6.75 6.62 6.60 6.55 6.53
5.19 5.17
3.92 3.89
1.61
1.12 1.00 1.00 0.96 0.96 1.08 1.02 1.06 0.95 1.00
1.06
3.00 3.04
5.90
f1 (ppm)

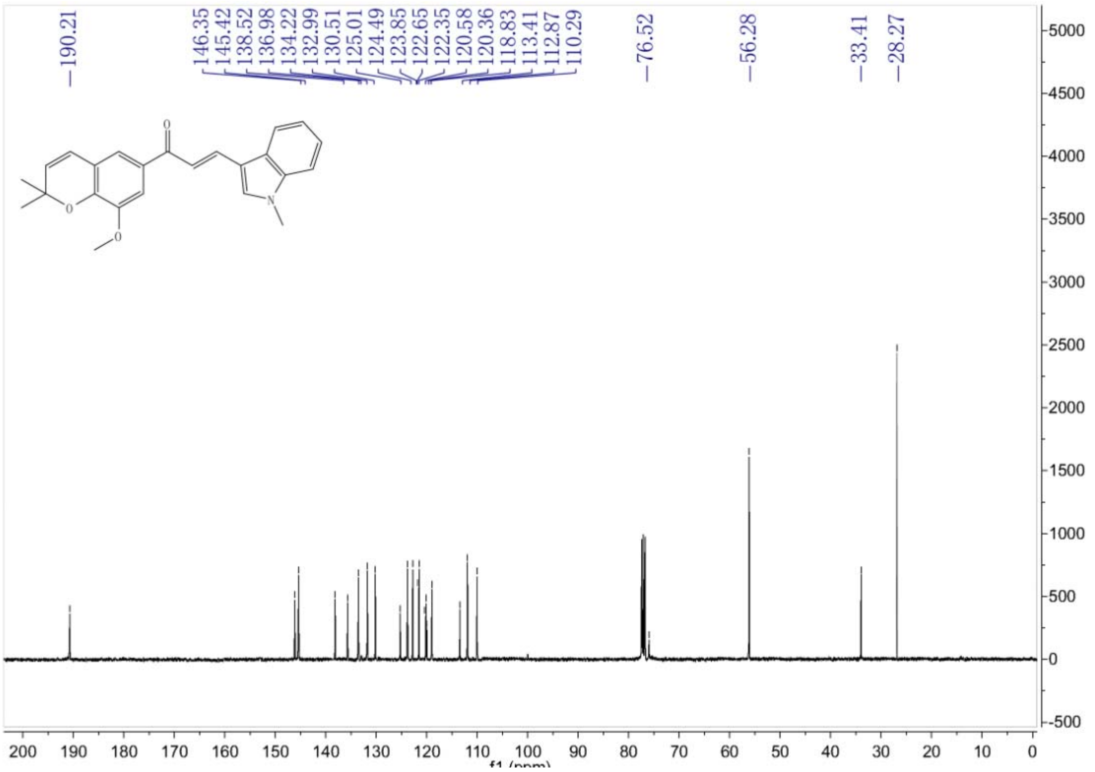
190.21
146.35 145.42 138.52 136.98 134.22 132.99 130.51 125.01 124.49 123.85 122.65 122.35 120.58 120.36 118.83 113.41 112.87 110.29
76.52
56.28
33.41
28.27

11a

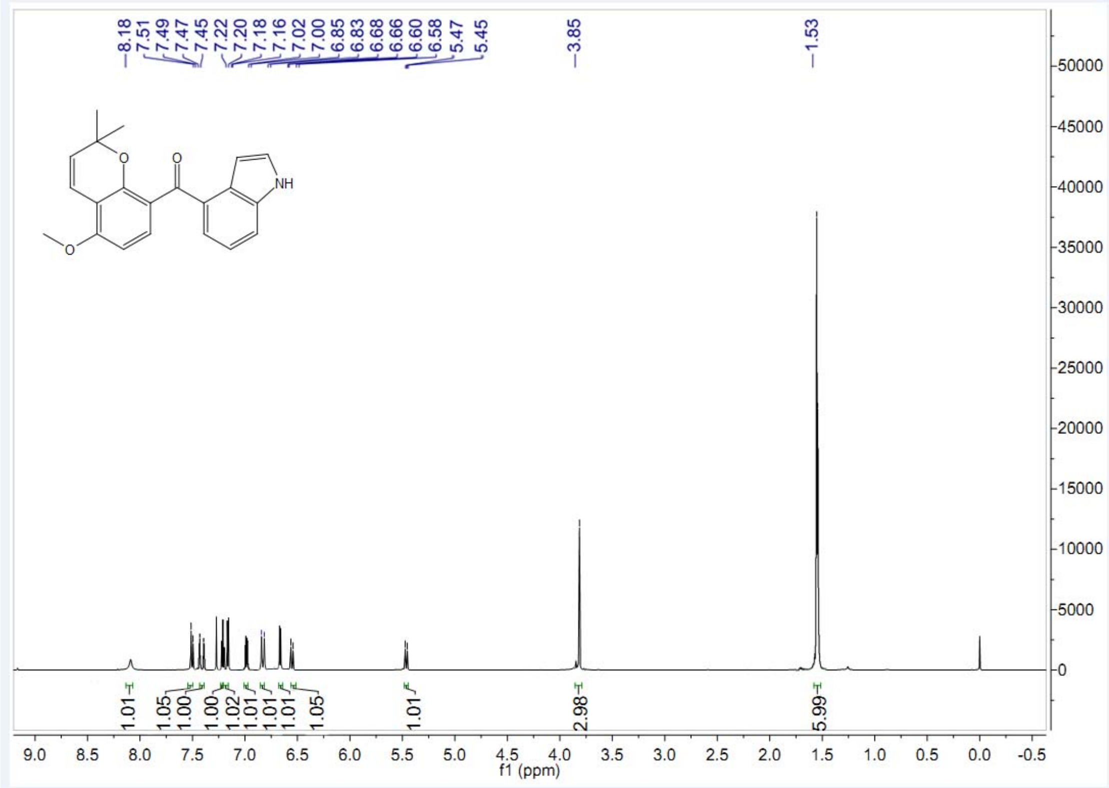
8.18
7.51
7.49
7.47
7.45
7.22
7.20
7.18
7.16
7.02
7.00
6.85
6.83
6.68
6.66
6.60
6.58
5.47
5.45
3.85
1.53
O
O
NH
O
1.01
1.05
1.00
1.00
1.02
1.01
1.01
1.01
1.05
1.01
2.98
5.99
f1 (ppm)

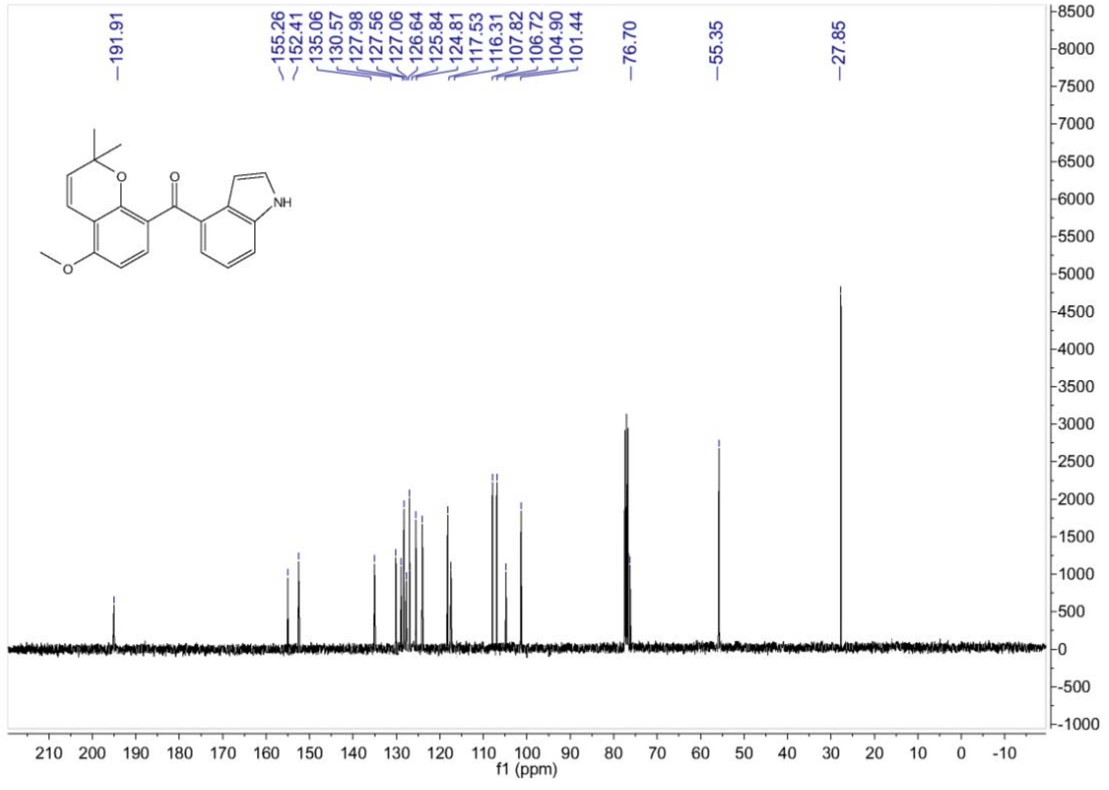
191.91
155.26
152.41
135.06
130.57
127.98
127.56
127.06
126.64
125.84
124.81
117.53
116.31
107.82
106.72
104.90
101.44
76.70
55.35
27.85
O
O
NH
O
f1 (ppm)

11b

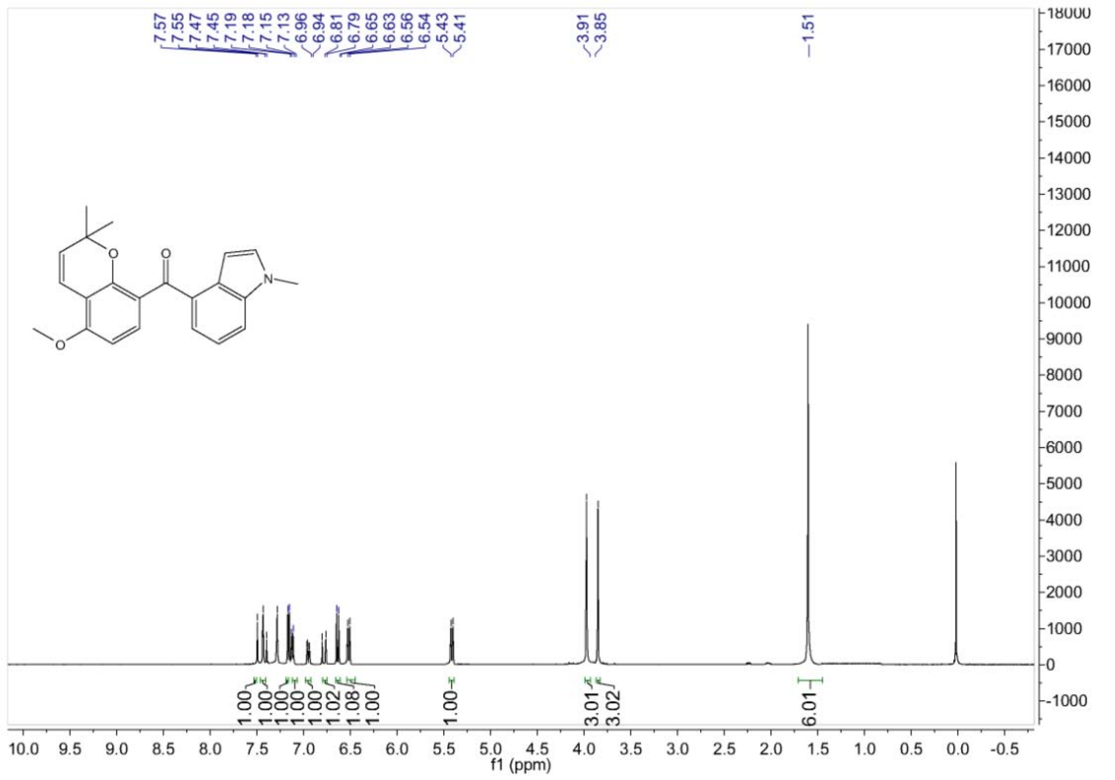
7.57 7.55 7.47 7.45 7.19 7.18 7.15 7.13 6.96 6.94 6.81 6.79 6.65 6.63 6.56 6.54 5.43 5.41
3.91 3.85
1.51
1.00 1.00 1.00 1.00 1.00 1.02 1.08 1.00
1.00
3.01 3.02
6.01
f1 (ppm)

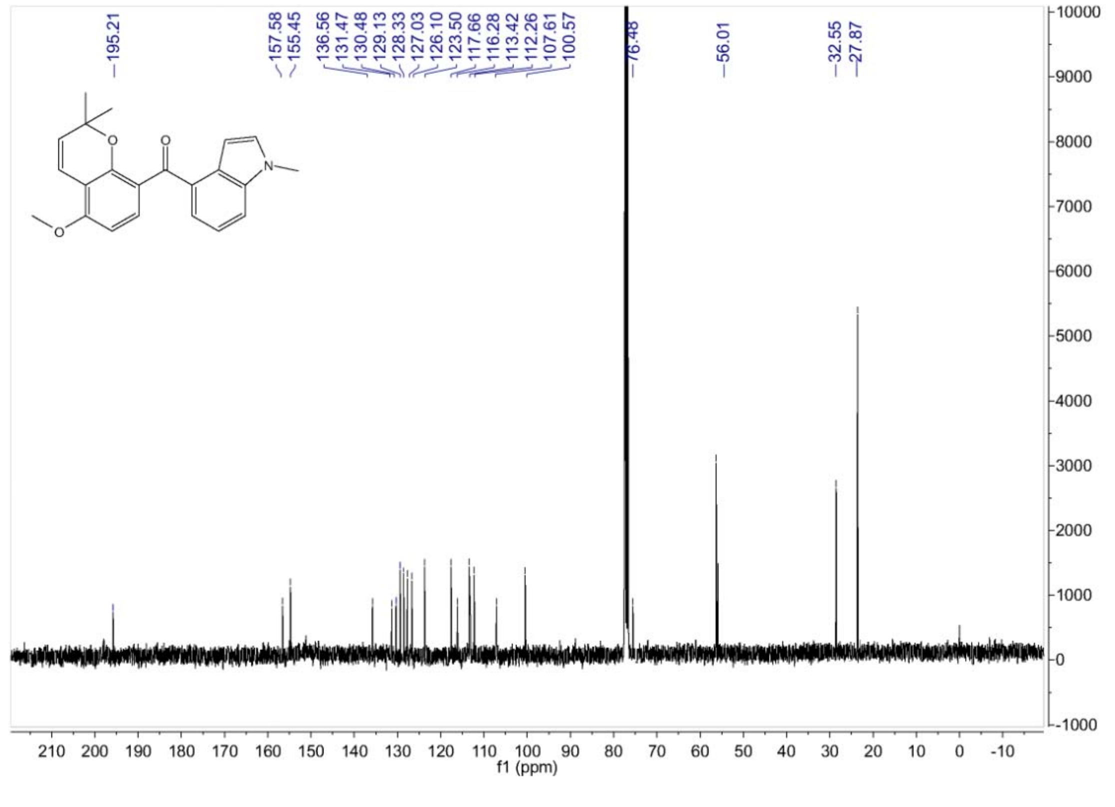
195.21
157.58 155.45
136.56 131.47 130.48 129.13 128.33 127.03 126.10 123.50 117.66 116.28 113.42 112.26 107.61 100.57
76.48
56.01
32.55 27.87
f1 (ppm)

14a

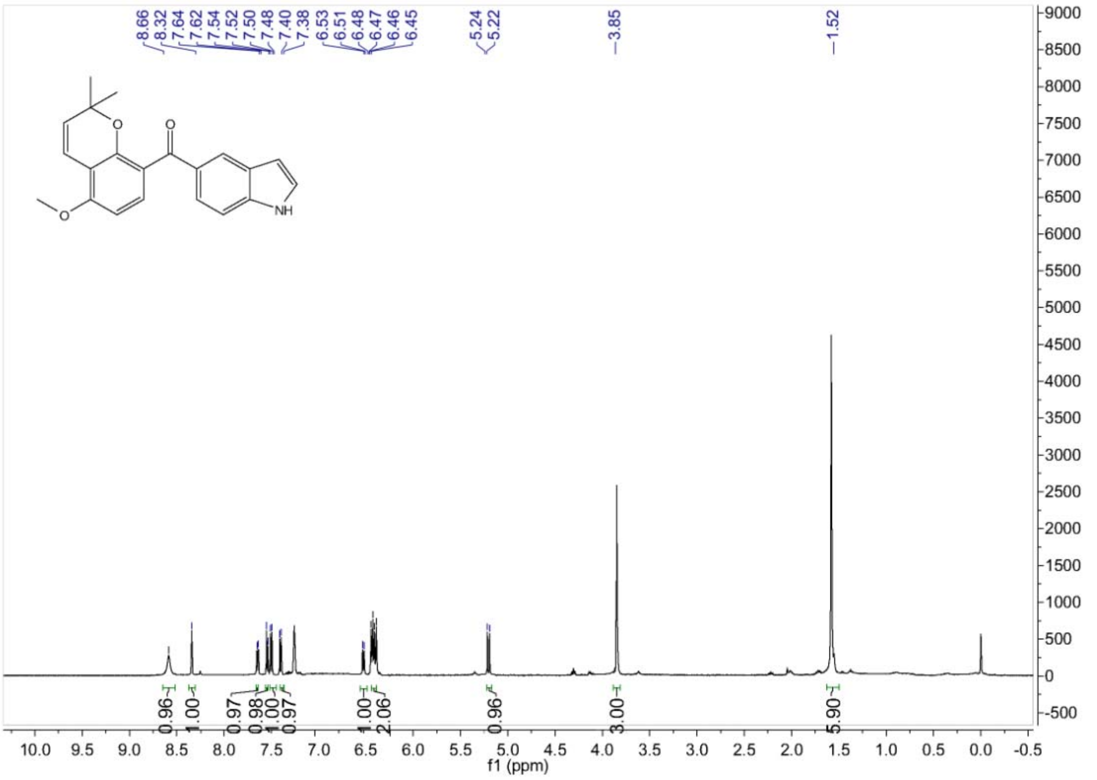
8.66
8.32
7.64
7.62
7.54
7.52
7.50
7.48
7.40
7.38
6.53
6.51
6.48
6.47
6.46
6.45
5.24
5.22
3.85
1.52
NH
O
0.96
1.00
0.97
0.98
1.00
0.97
1.00
2.06
0.96
3.00
5.90
f1 (ppm)

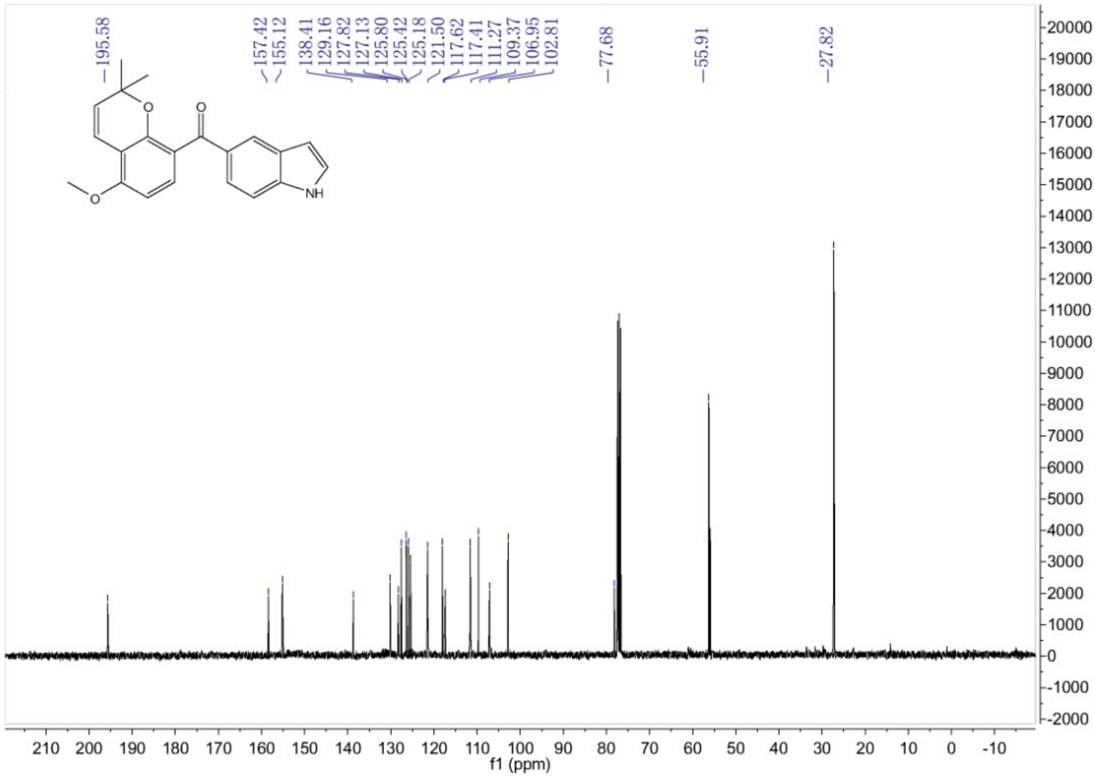
195.58
157.42
155.12
138.41
129.16
127.82
127.13
125.80
125.42
125.18
121.50
117.62
117.41
111.27
109.37
106.95
102.81
77.68
55.91
27.82
NH
O
f1 (ppm)

14b

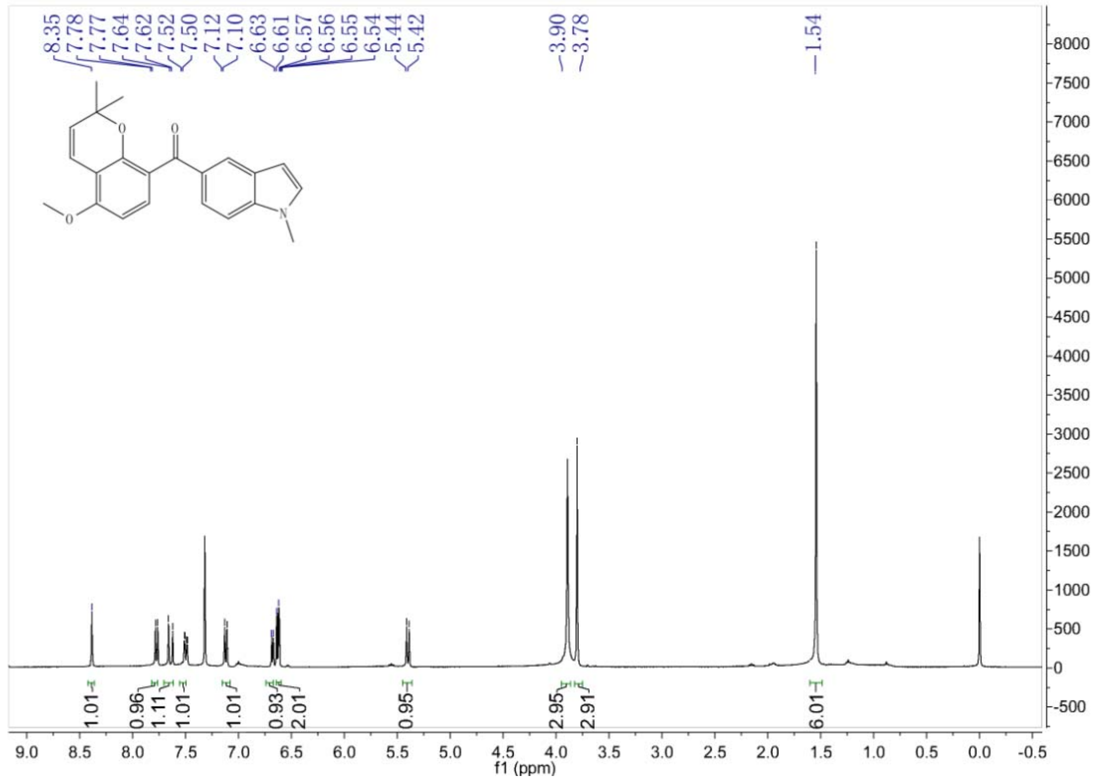
8.35
7.78
7.77
7.64
7.62
7.52
7.50
7.12
7.10
6.63
6.61
6.57
6.56
6.55
6.54
5.44
5.42
3.90
3.78
1.54
1.01
0.96
1.11
1.01
1.01
0.93
2.01
0.95
2.95
2.91
6.01
f1 (ppm)

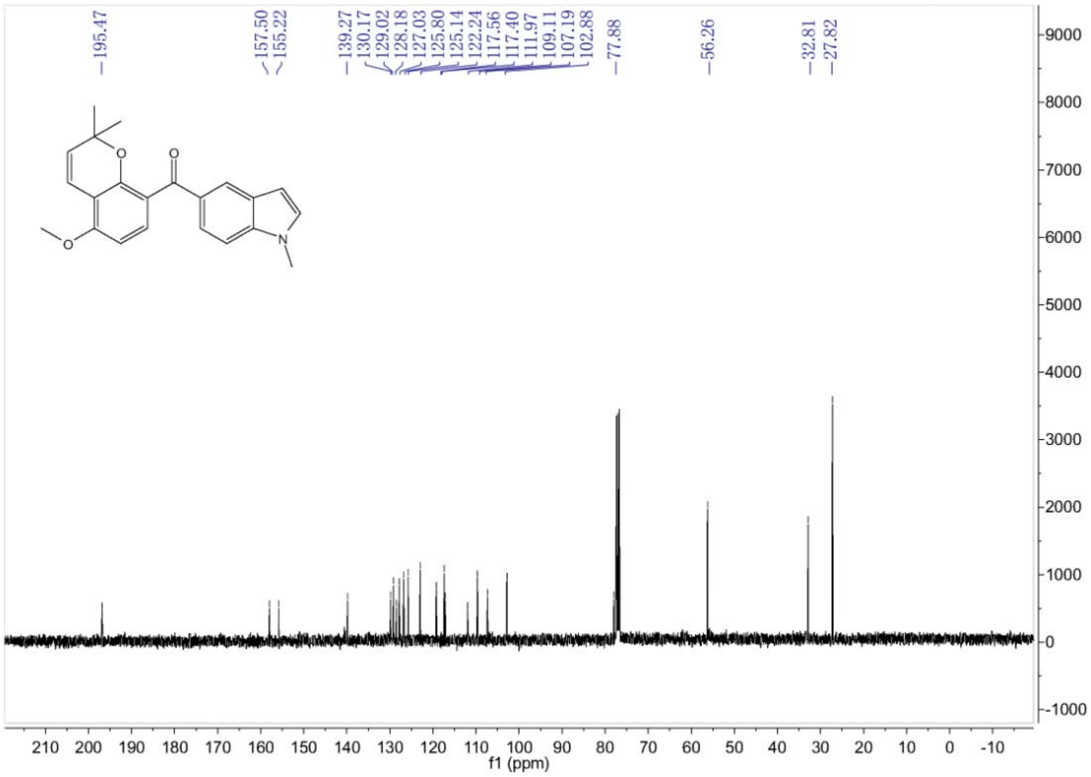
195.47
157.50
155.22
139.27
130.17
129.02
128.18
127.03
125.80
125.14
122.24
117.56
117.40
111.97
109.11
107.19
102.88
77.88
56.26
32.81
27.82
f1 (ppm)

19a

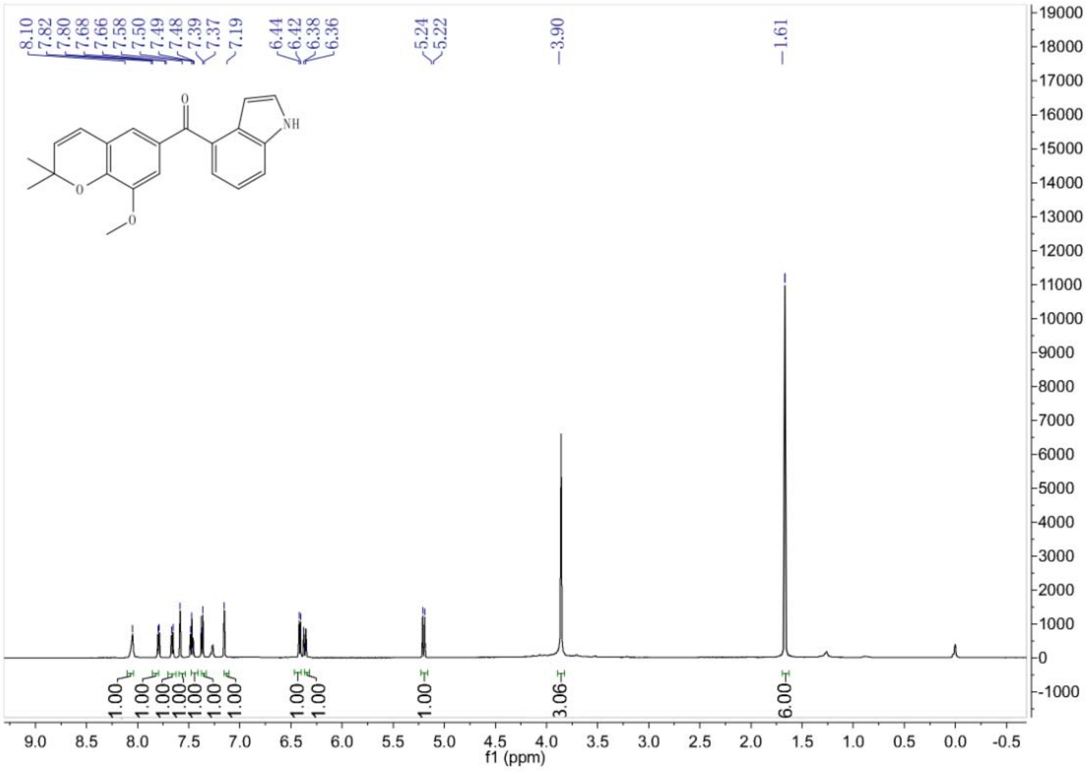

8.10
7.82
7.80
7.68
7.66
7.58
7.50
7.49
7.48
7.39
7.37
7.19
6.44
6.42
6.38
6.36
5.24
5.22
3.90
1.61
NH
O
1.00
1.00
1.00
1.00
1.00
1.00
1.00
1.00
1.00
3.06
6.00
f1 (ppm)

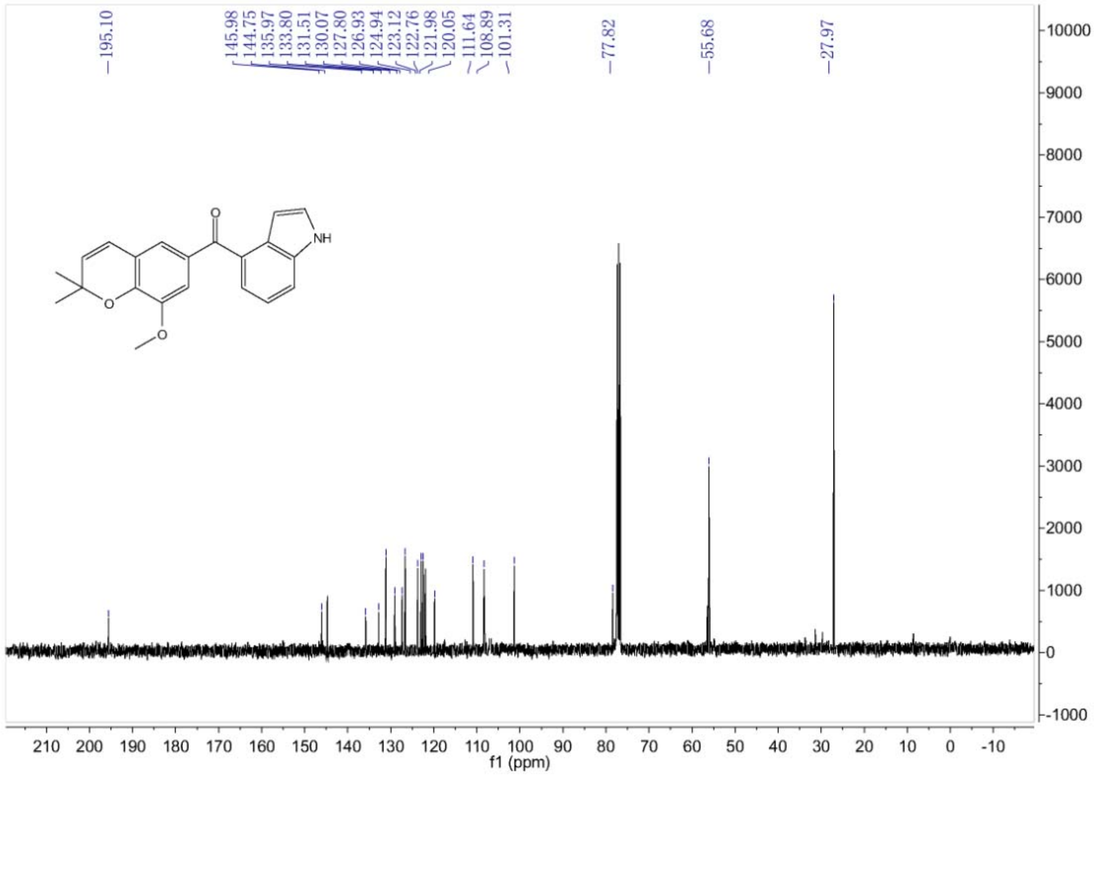

195.10
145.98
144.75
135.97
133.80
131.51
130.07
127.80
126.93
124.94
123.12
122.76
121.98
120.05
111.64
108.89
101.31
77.82
55.68
27.97
NH
O
f1 (ppm)

19b

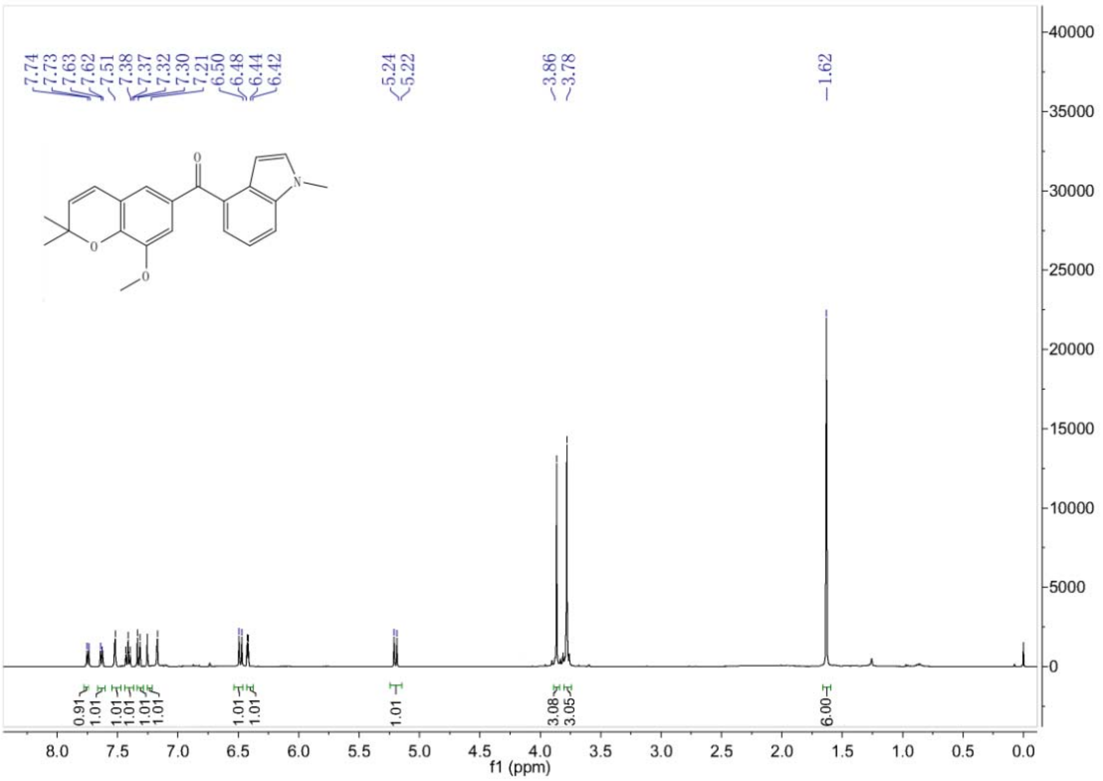
7.74
7.73
7.63
7.62
7.51
7.38
7.37
7.32
7.30
7.21
6.50
6.48
6.44
6.42
5.24
5.22
3.86
3.78
1.62
O
N
O
O
0.91
1.01
1.01
1.01
1.01
1.01
1.01
1.01
1.01
3.08
3.05
6.00
f1 (ppm)

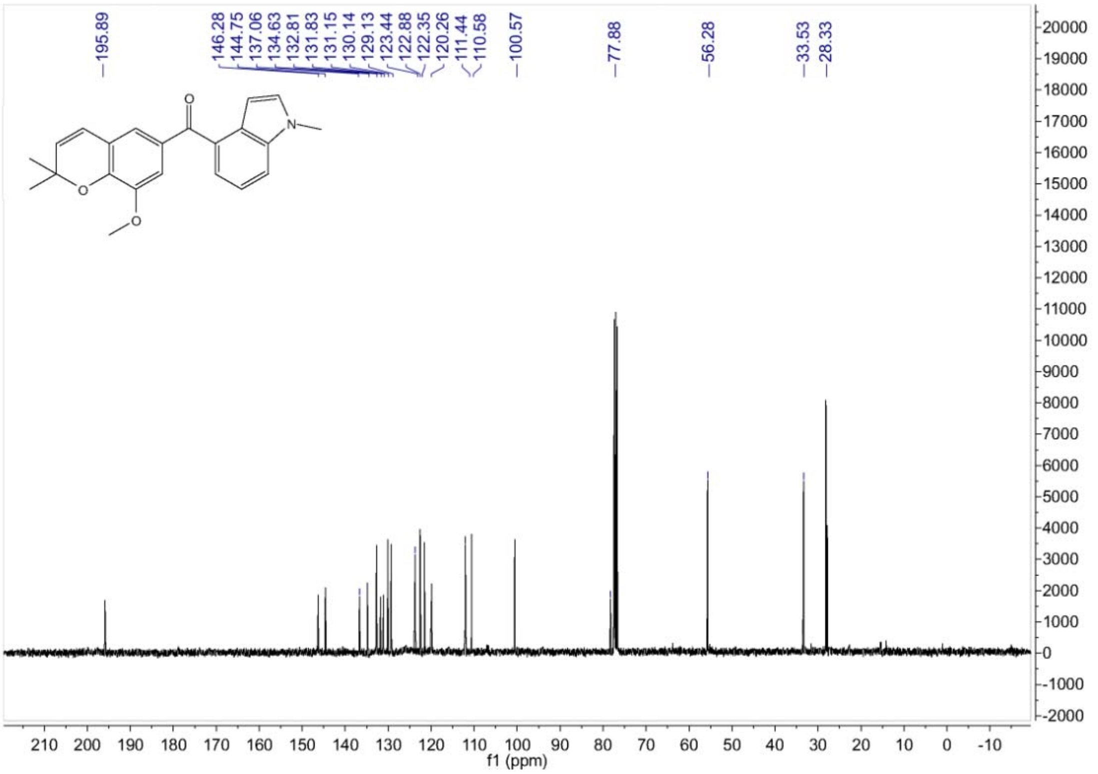
195.89
146.28
144.75
137.06
134.63
132.81
131.83
131.15
130.14
129.13
123.44
122.88
122.35
120.26
111.44
110.58
100.57
77.88
56.28
33.53
28.33
O
N
O
O
f1 (ppm)

21a

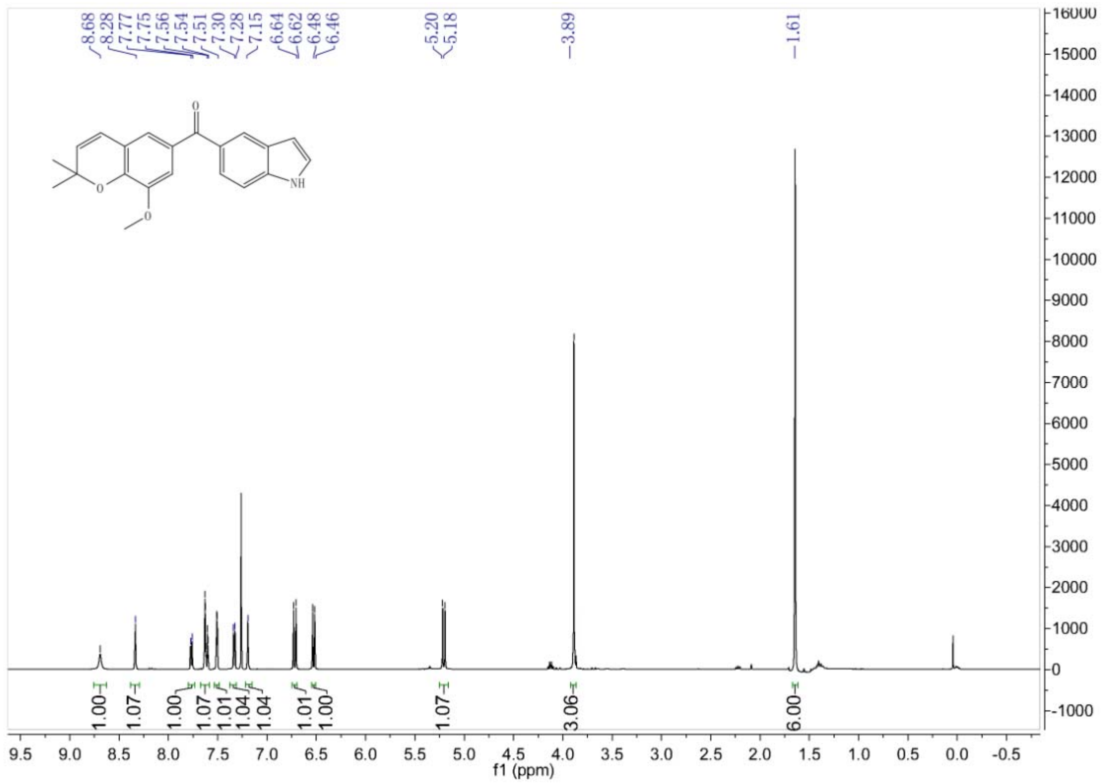
8.68
8.28
7.77
7.75
7.56
7.54
7.51
7.30
7.28
7.15
6.64
6.62
6.48
6.46
5.20
5.18
3.89
1.61
NH
O
1.00
1.07
1.00
1.07
1.01
1.04
1.04
1.01
1.00
1.07
3.06
6.00
f1 (ppm)

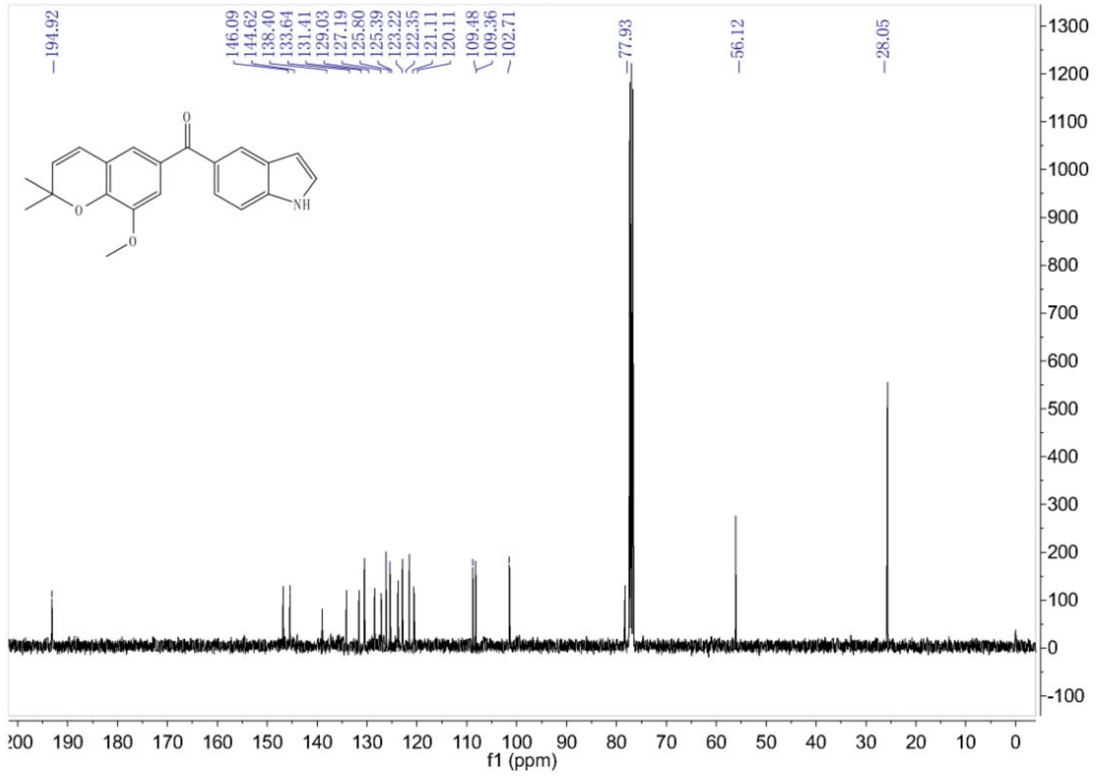
194.92
146.09
144.62
138.40
133.64
131.41
129.03
127.19
125.80
125.39
123.22
122.35
121.11
120.11
109.48
109.36
102.71
77.93
56.12
28.05
NH
O
f1 (ppm)

21b

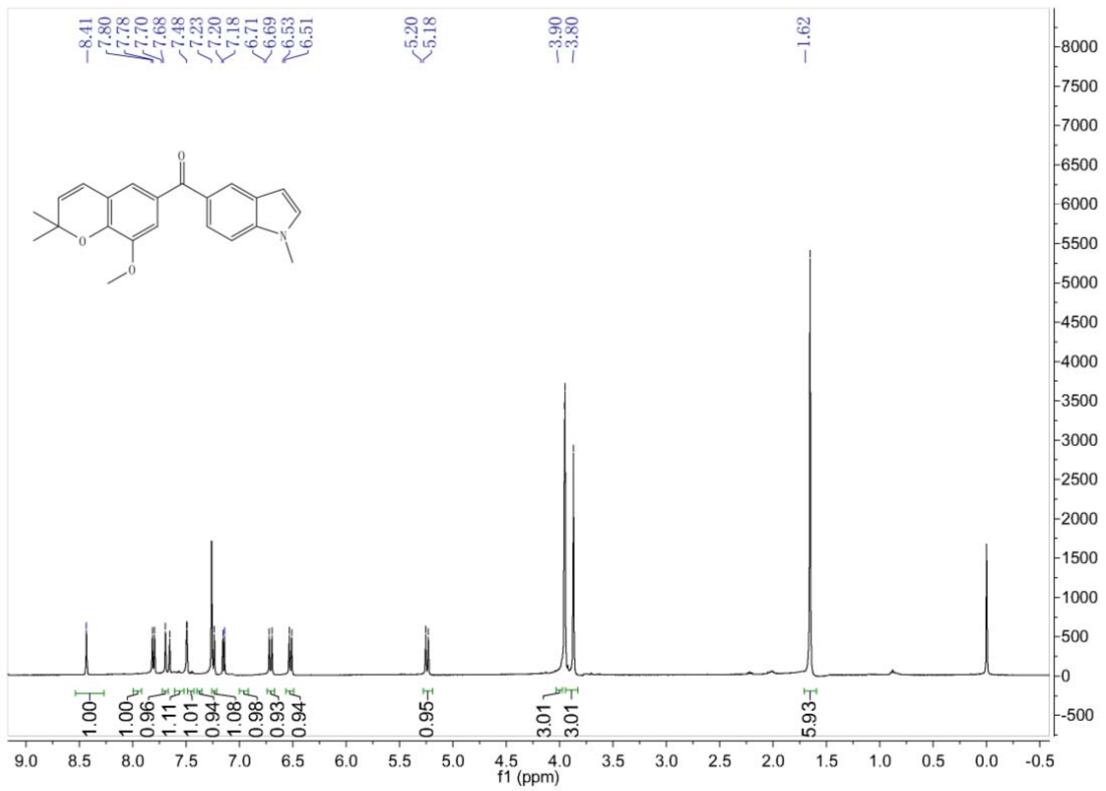

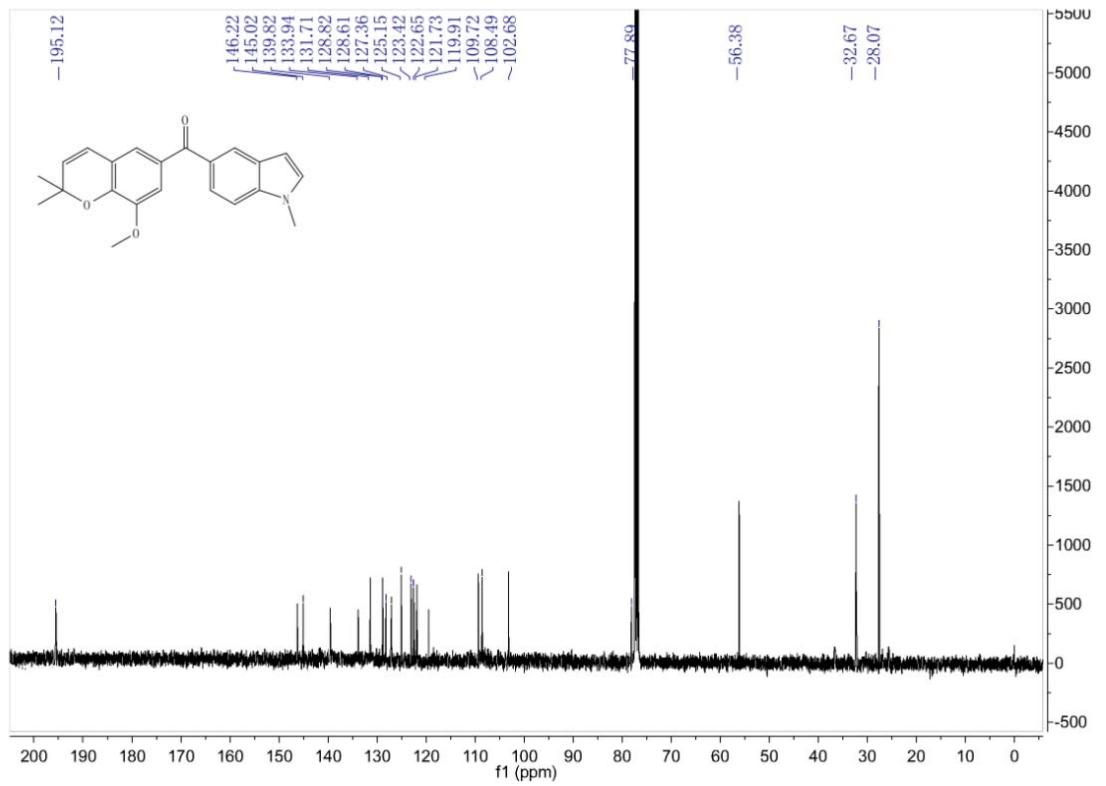

S II. HPLC chromatograms of target compounds

5a

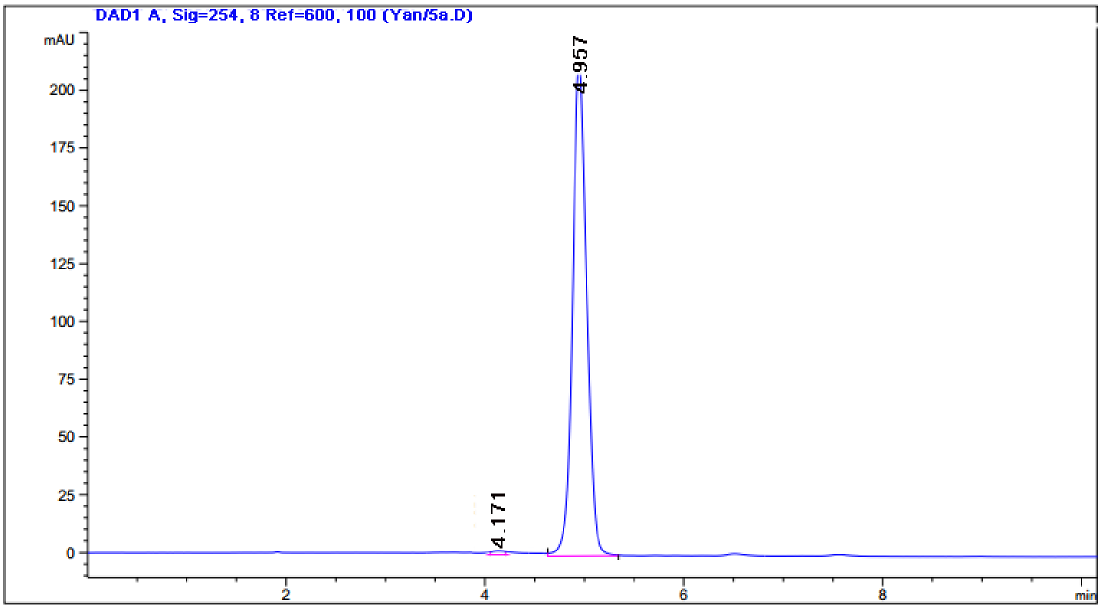

Ret.Time	Compounds	Height [mAU]	Area [mAU*S]	Area%
4.171		6.38015	50.02044	2.2875
4.957	5a	222.02824	2136.60725	97.7215

5b

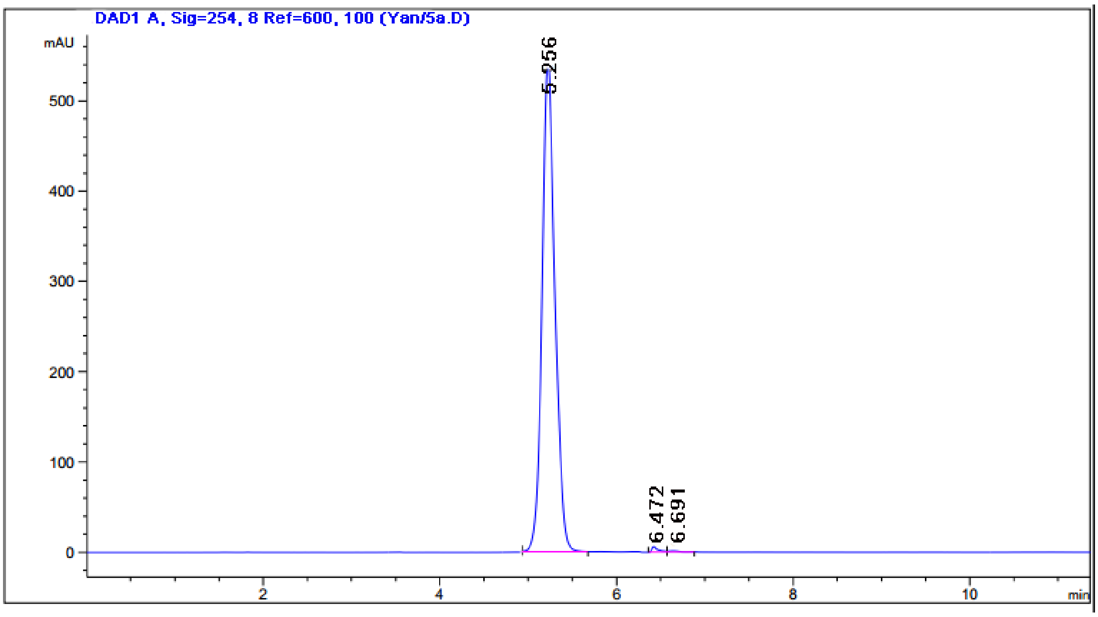

Ret.Time	Compounds	Height [mAU]	Area [mAU*S]	Area%
5.256	5b	548.26951	5802.87790	99.1592
6.472		6.16198	32.61144	0.5876
6.691		1.43769	14.04958	0.2532

11a

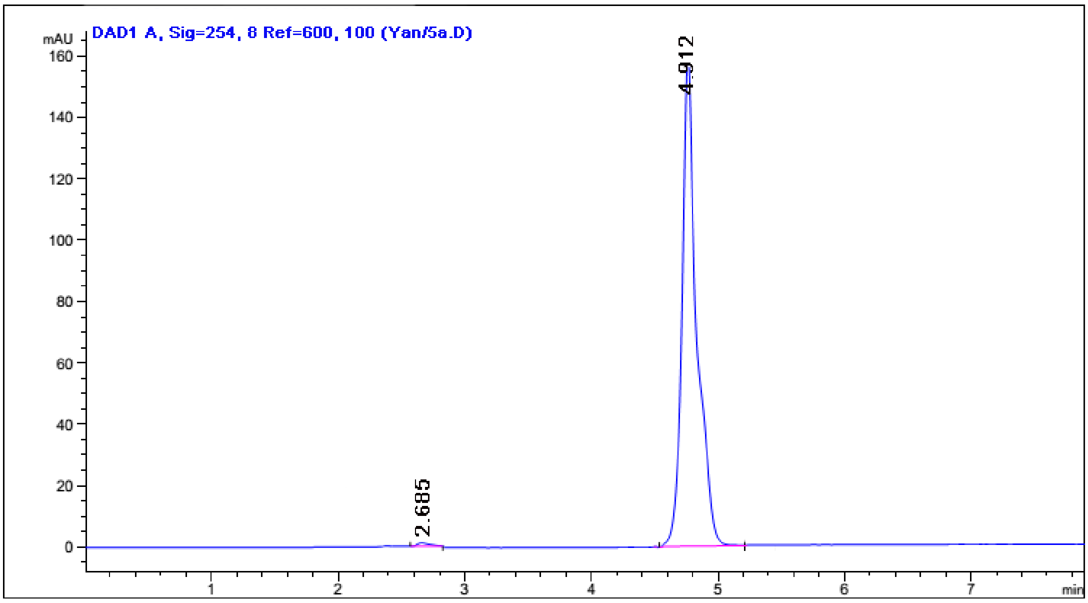

Ret.Time	Compounds	Height [mAU]	Area [mAU*S]	Area%
2.685		18.25157	14.56981	1.0744
4.912	11a	158.62542	1202.23598	98.9256

11b

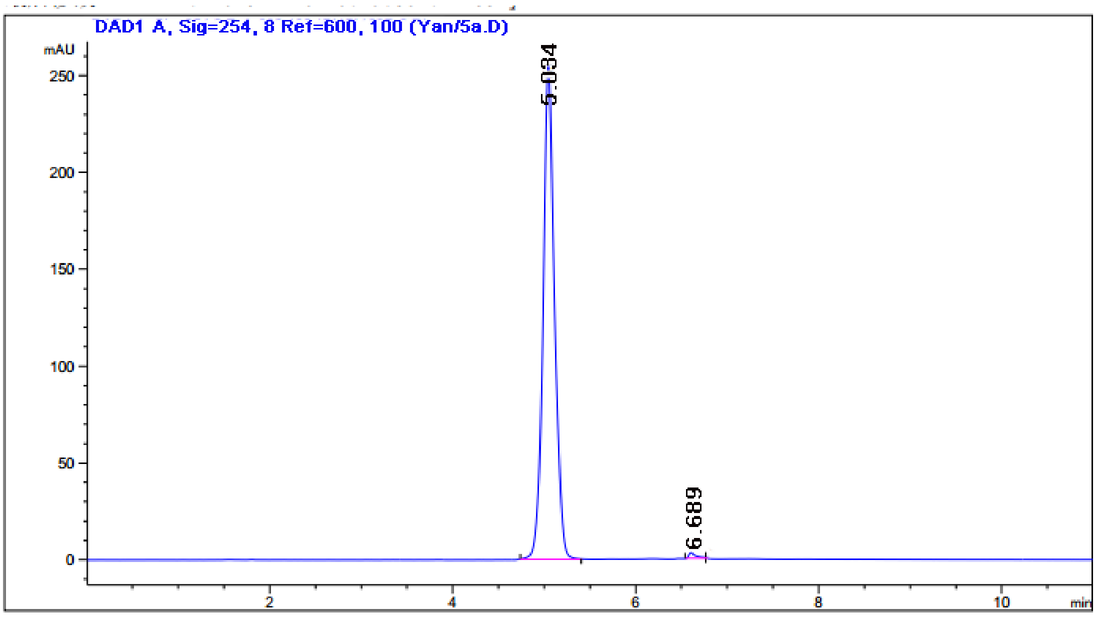

Ret.Time	Compounds	Height [mAU]	Area [mAU*S]	Area%
5.034	11b	254.63928	2218.95093	99.1829
6.689		2.30604	18.28127	0.8171

14a

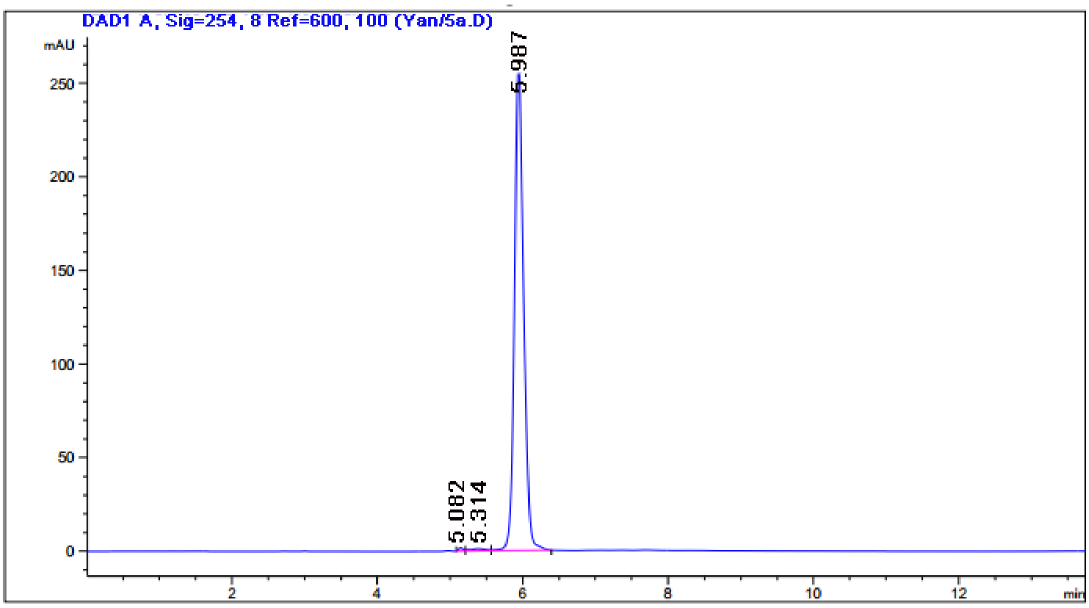

Ret.Time	Compounds	Height [mAU]	Area [mAU*S]	Area%
5.082		2.11247	8.62158	0.3762
5.314		1.36164	21.92517	0.9563
5.987	14a	261.61893	2267.94828	98.6675

14b

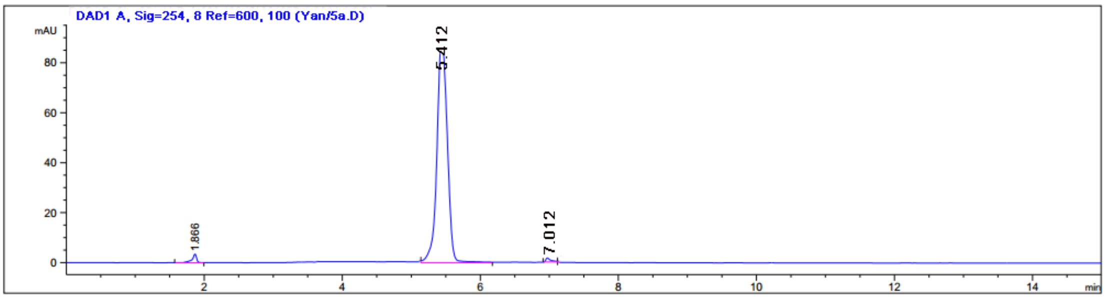

Ret.Time	Compounds	Height [mAU]	Area [mAU*S]	Area%
1.866		3.43061	15.82136	1.6254
5.412	14b	90.80245	935.26545	96.6251
7.012		3.21025	21.52102	1.7495

19a

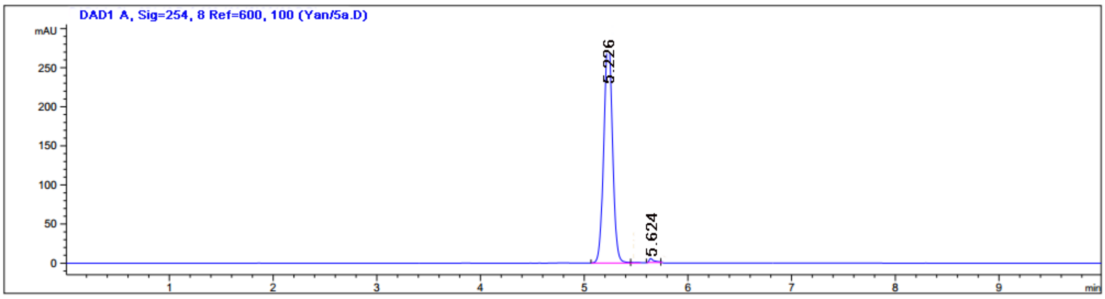

Ret.Time	Compounds	Height [mAU]	Area [mAU*S]	Area%
5.226	19a	290.89590	1663.47725	98.2219
5.624		4.10718	29.71801	1.7781

19b

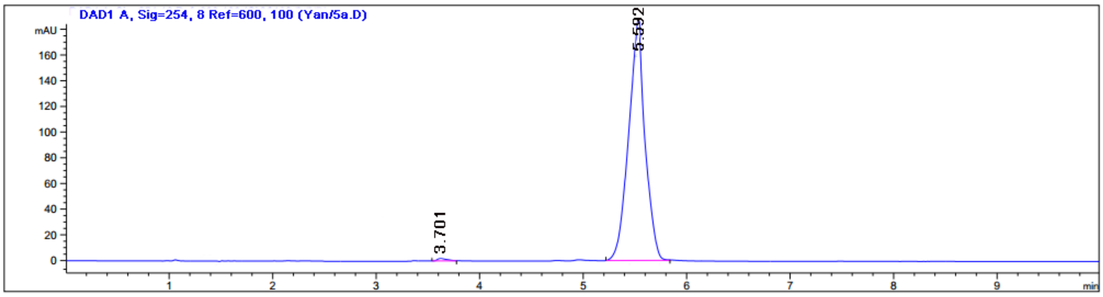

Ret.Time	Compounds	Height [mAU]	Area [mAU*S]	Area%
3.701		13.89284	146.58921	2.1256
5.592	19b	175.31985	4052.98219	97.8744

21a

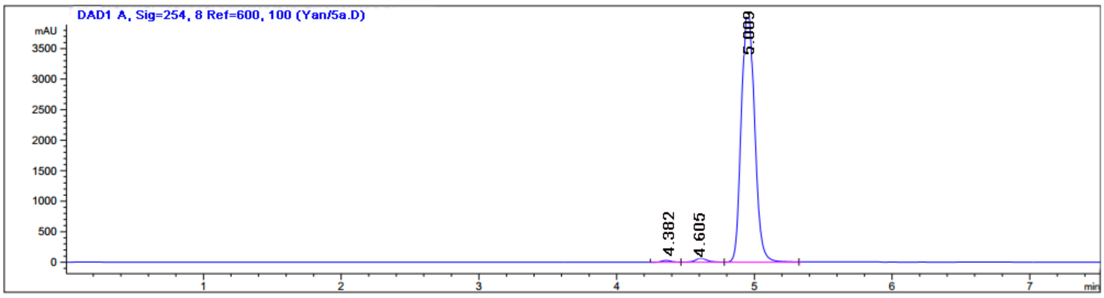

Ret.Time	Compounds	Height [mAU]	Area [mAU*S]	Area%
4.382		29.80025	153.20251	0.5577
4.605		63.88231	400.24856	1.4595
5.009	21a	3724.65981	26895.20325	97.9828

21b

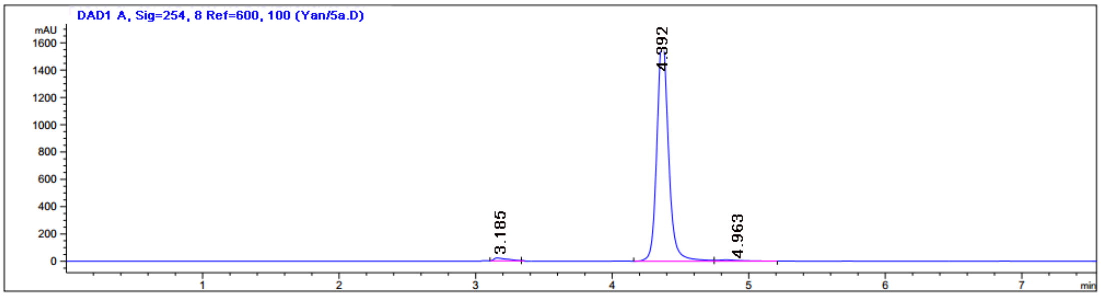

Ret.Time	Compounds	Height [mAU]	Area [mAU*S]	Area%
3.185		5.26241	59.05641	0.6015
4.392	21b	1660.65039	9681.35742	98.1793
4.963		10.58348	119.49019	1.2192

SⅢ.High resolution mass spectra of target compounds

5a

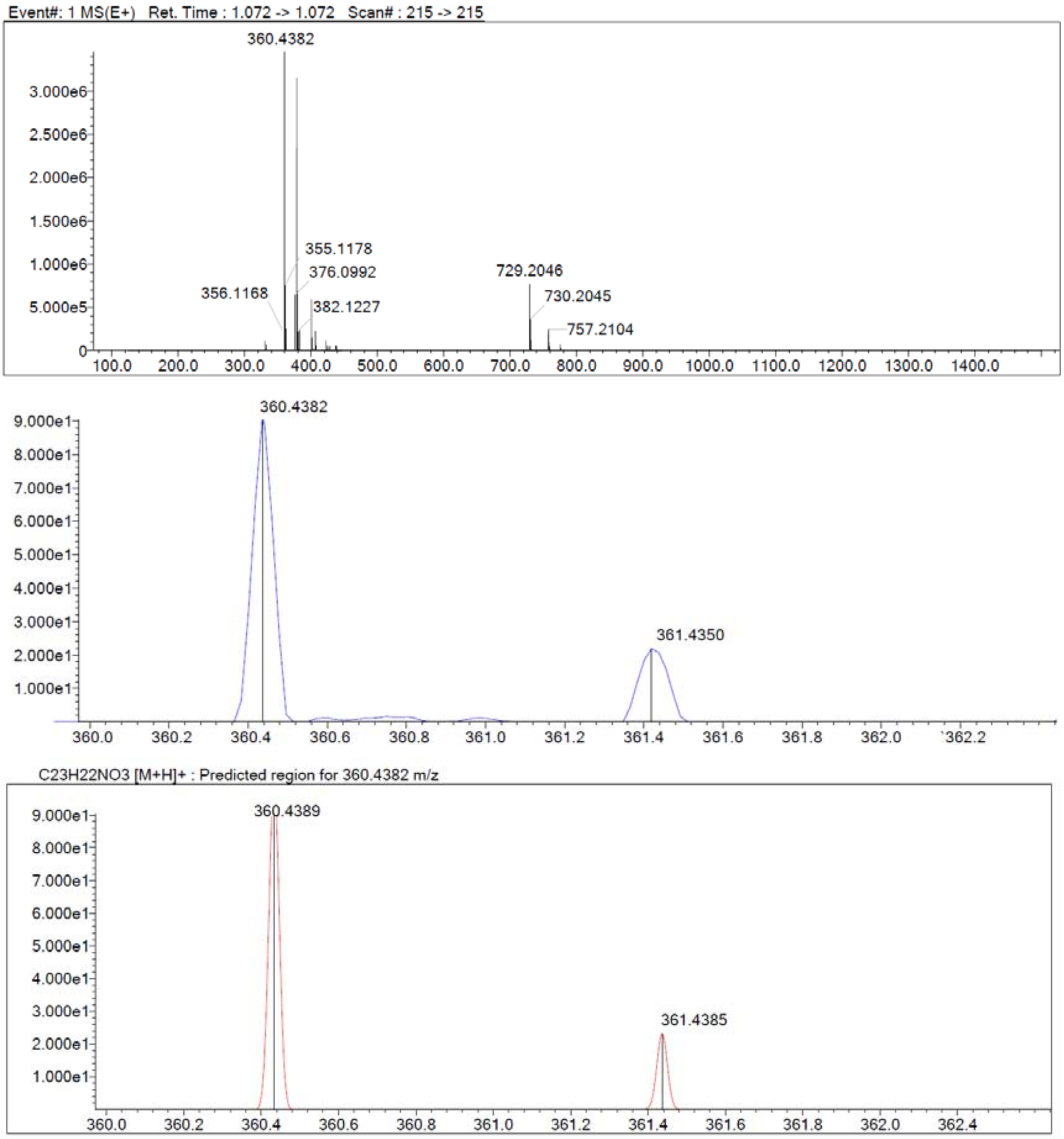

Rank	Score	Formula (M)	Ion	Meas. m/z	Pred. m/z	Df. (mDa)	Df. (ppm)	Iso	DBE
1	78.93	C23H22NO3	[M+H]+	360.4382	360.4389	-0.7	-1.98	80.91	12.0

5b

Event#: 1 MS(E+) Ret. Time : 0.765 -> 0.765 Scan# : 154 -> 154

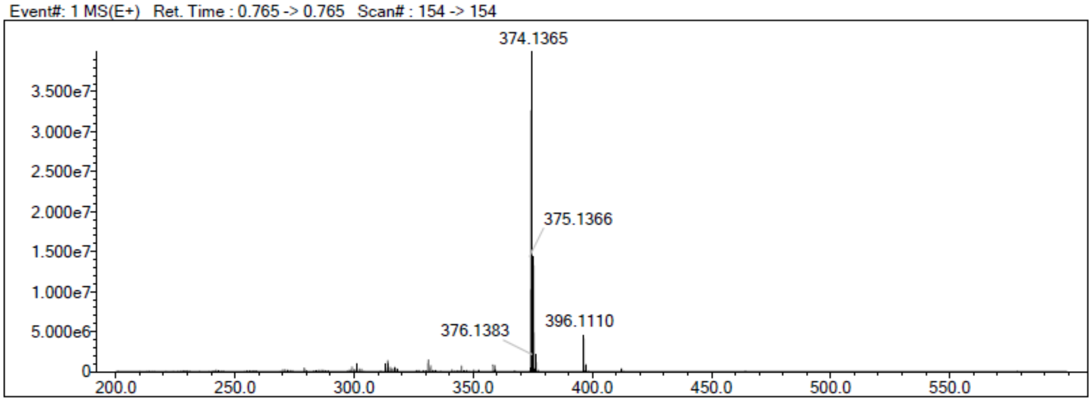

Measured region for 374.1365 m/z

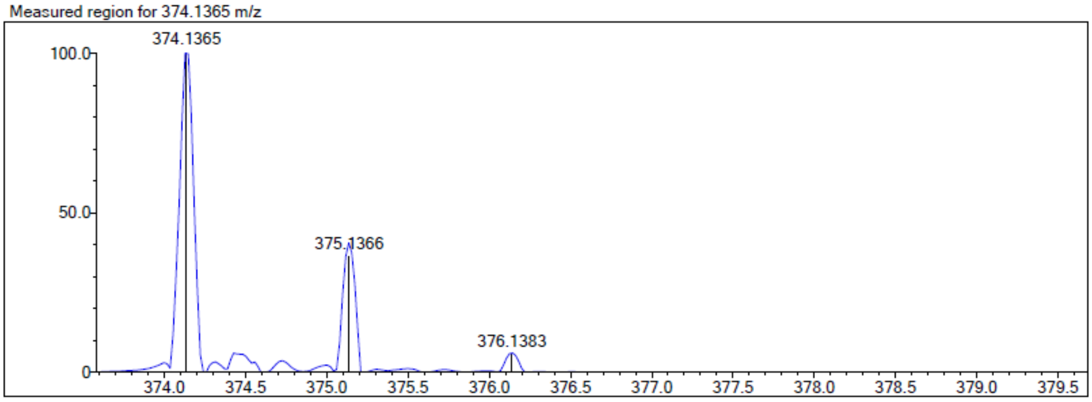

C24 H24 N O3 [M+H]+ : Predicted region for 374.1351 m/z

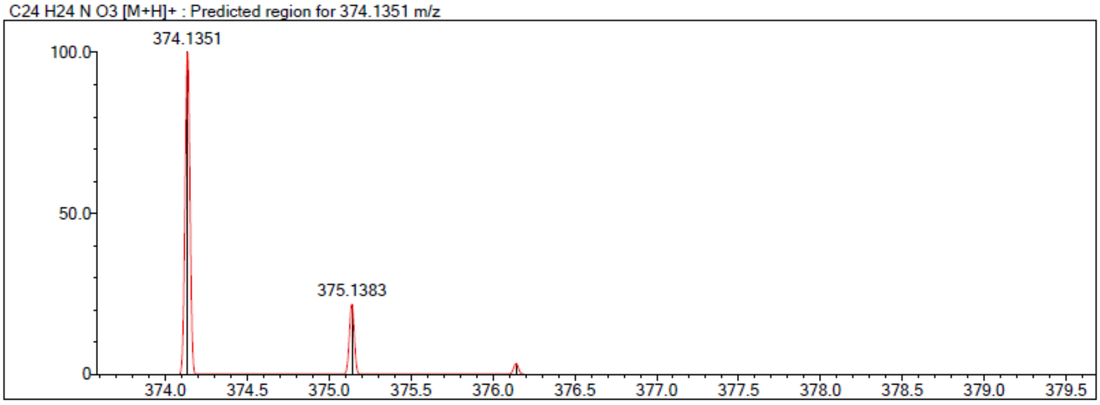

Rank	Score	Formula (M)	Ion	Meas. m/z	Pred. m/z	Df. (mDa)	Df. (ppm)	Iso	DBE
10	43.69	C24 H24 NO3	[M+H]+	374.1365	374.1351	1.4	3.73	46.89	11.0

11a

Event#: 1 MS(E+) Ret. Time : 0.932 Scan# : 187

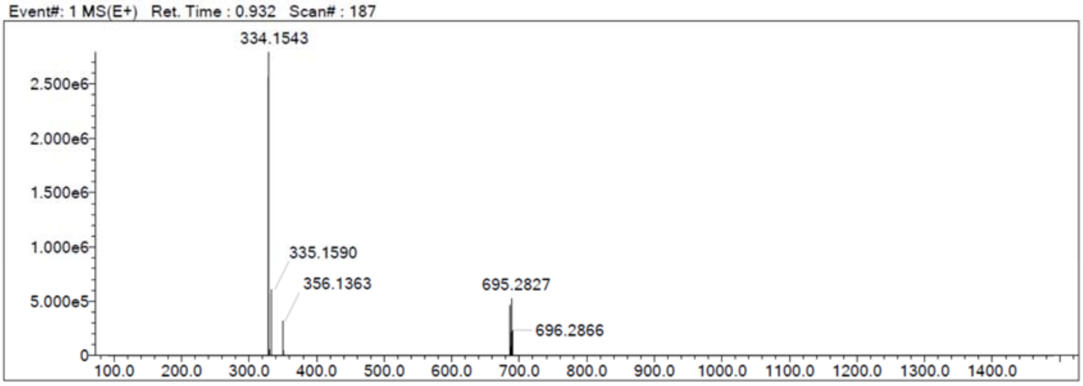

Measured region for 334.1543 m/z

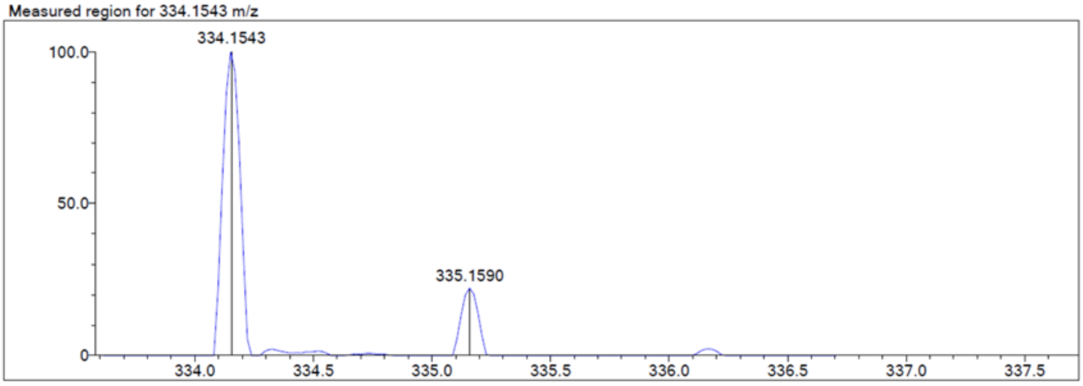

C21 H20 N O3 [M+H]+ : Predicted region for 334.1547 m/z

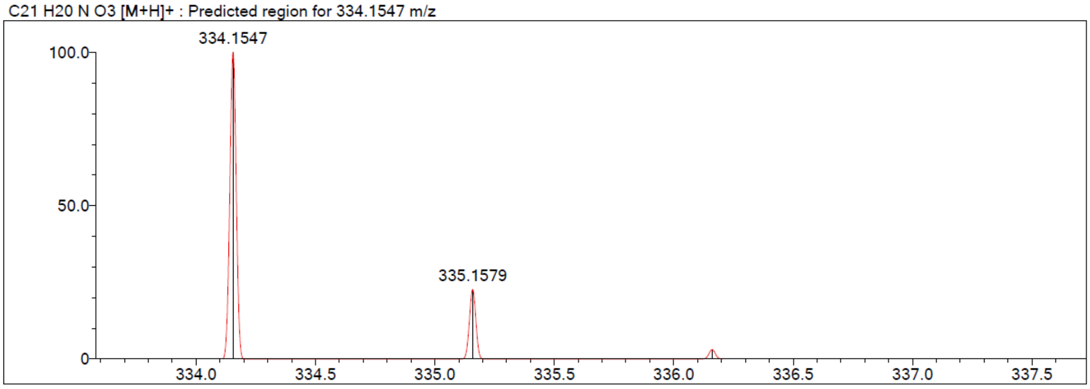

Rank	Score	Formula (M)	Ion	Meas. m/z	Pred. m/z	Df. (mDa)	Df. (ppm)	Iso	DBE
1	88.21	C21 H20 N O3	[M+H]+	334.1543	334.1547	-0.4	-1.19	88.64	12.0

11b

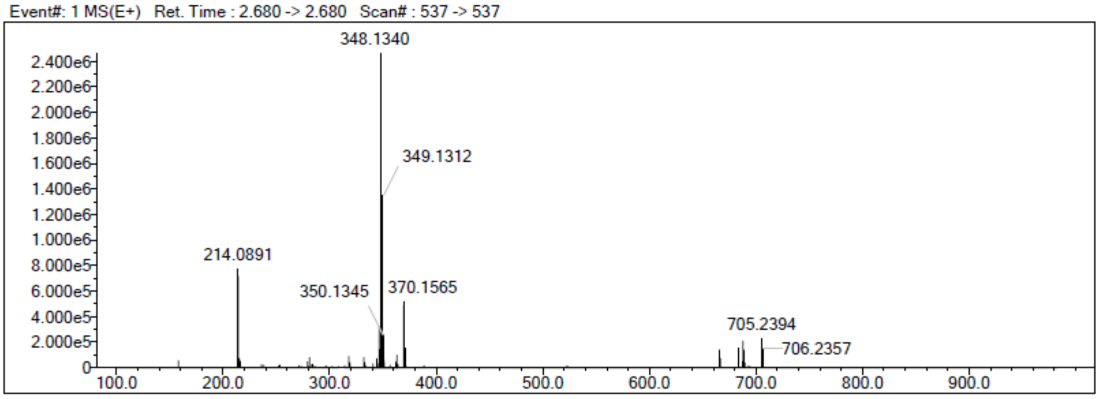

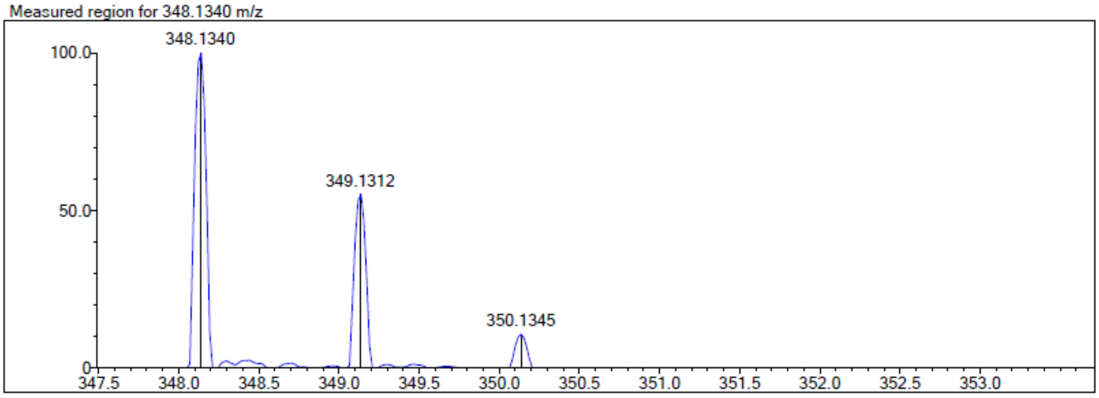

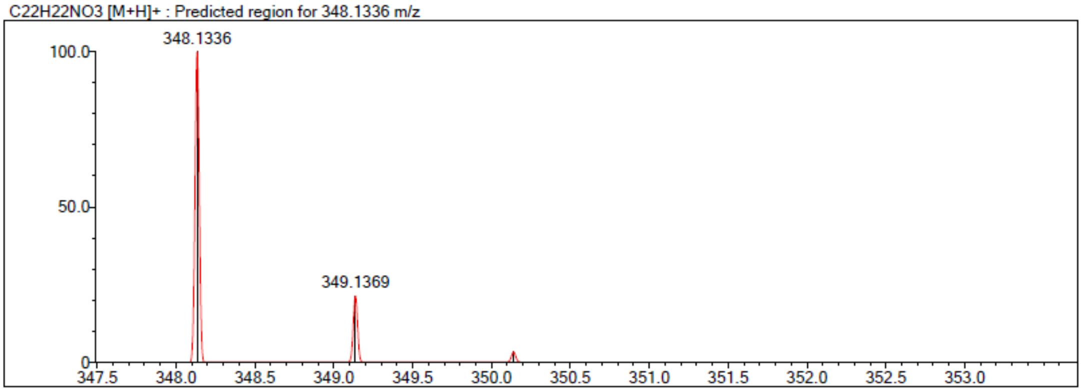

Rank	Score	Formula (M)	Ion	Meas. m/z	Pred. m/z	Df. (mDa)	Df. (ppm)	Iso	DBE
4	27.62	C22 H22 N O3	[M+H]+	348.1340	348.1336	0.4	1.17	27.74	11.0

14a

Event#: 1 MS(E+) Ret. Time : 0.840 -> 0.840 Scan# : 169 -> 169

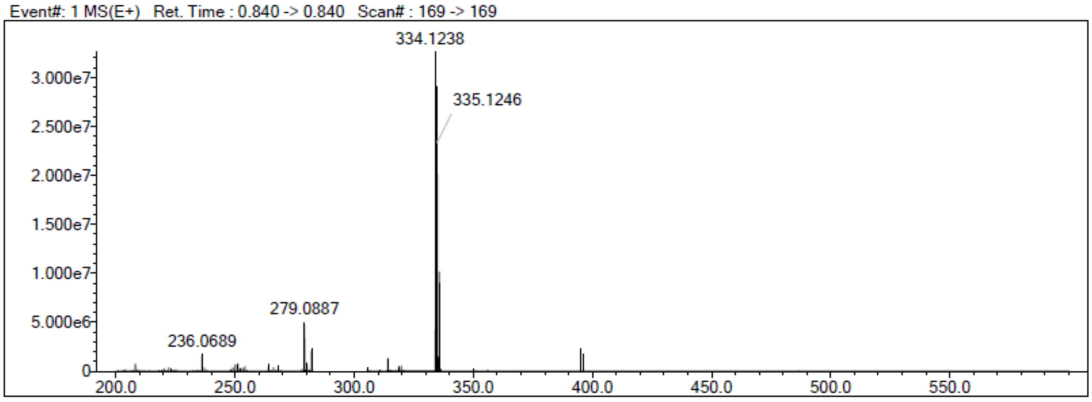

Measured region for 334.1238 m/z

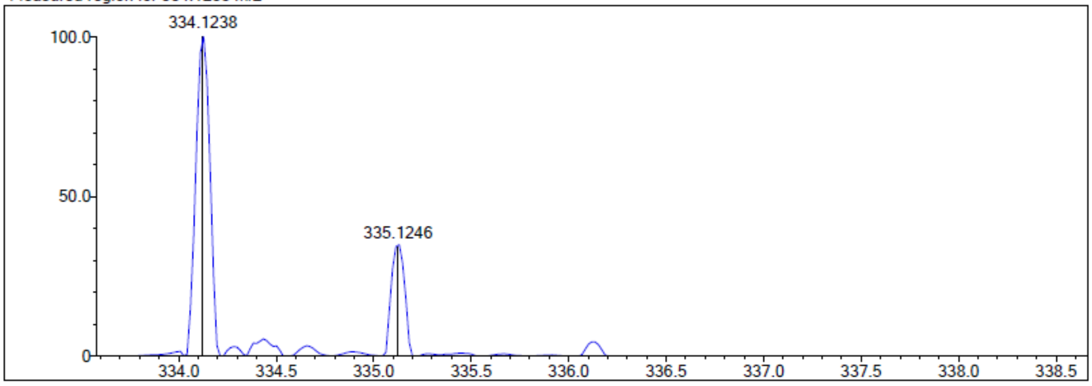

C21H20NO3 [M+H]+ : Predicted region for 334.1230 m/z

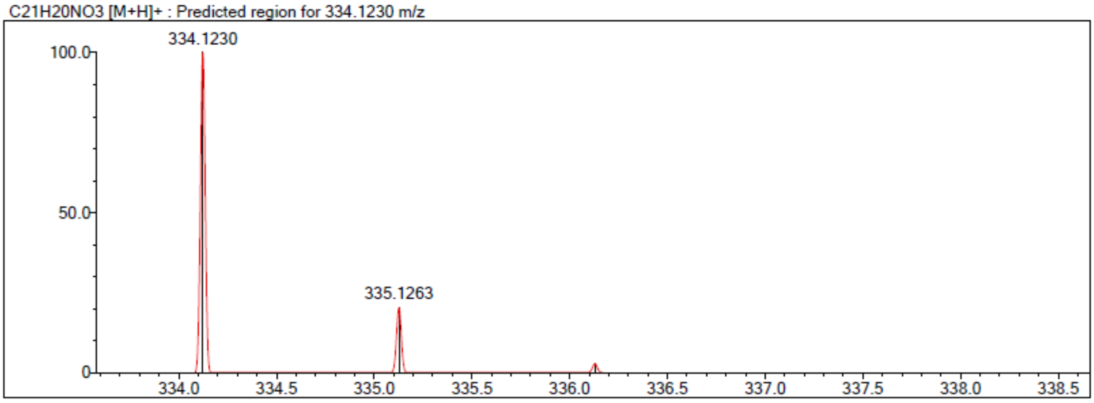

Rank	Score	Formula (M)	Ion	Meas. m/z	Pred. m/z	Df. (mDa)	Df. (ppm)	Iso	DBE
4	43.48	C21H20NO3	[M+H]+	334.1238	334.1230	0.8	2.56	45.24	11.0

14b

Event#: 1 MS(E+) Ret. Time : 0.840 -> 0.840 Scan# : 169 -> 169

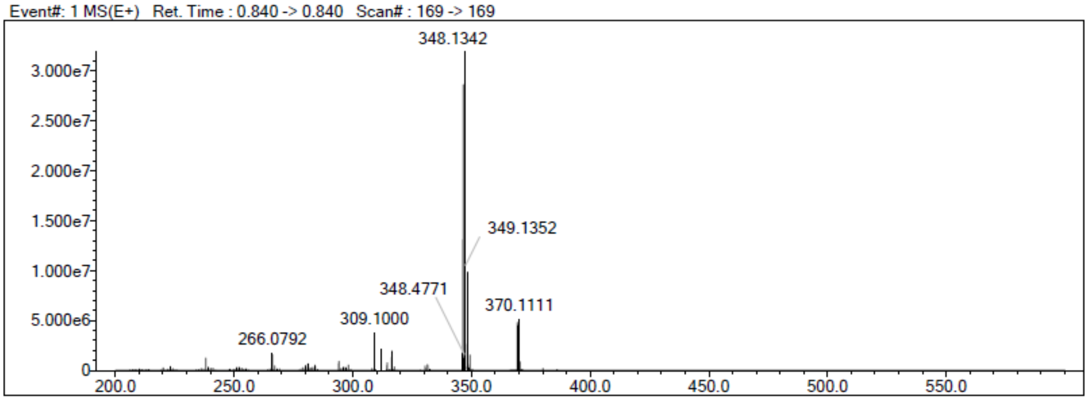

Measured region for 348.1342 m/z

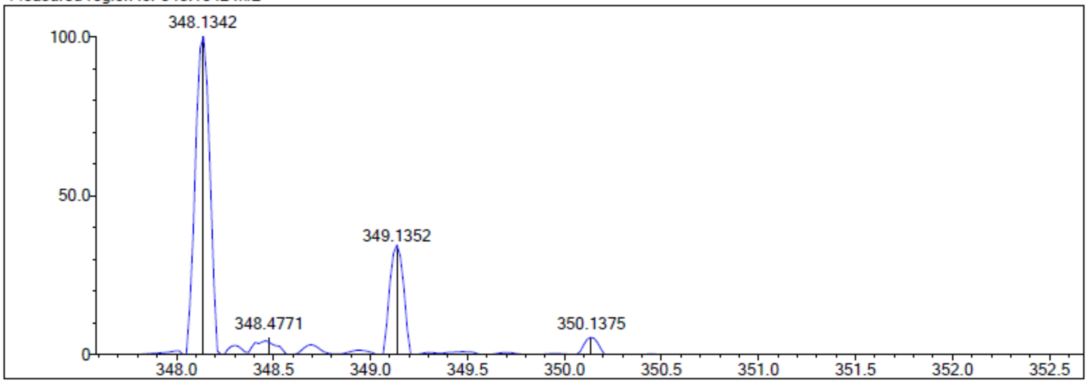

C22H22NO3 [M+H]+ : Predicted region for 348.1336 m/z

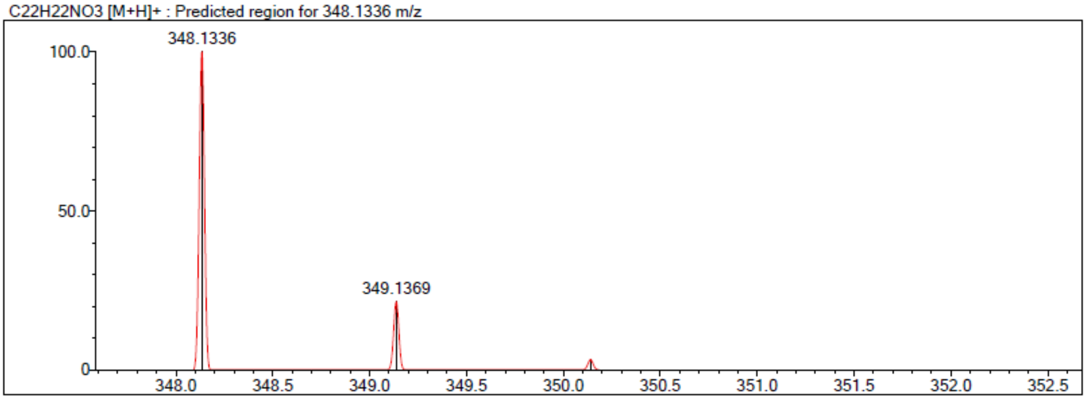

Rank	Score	Formula (M)	Ion	Meas. m/z	Pred. m/z	Df. (mDa)	Df. (ppm)	Iso	DBE
3	49.40	C22H22NO3	[M+H]+	348.1342	348.1336	0.6	1.75	50.35	11.0

19a

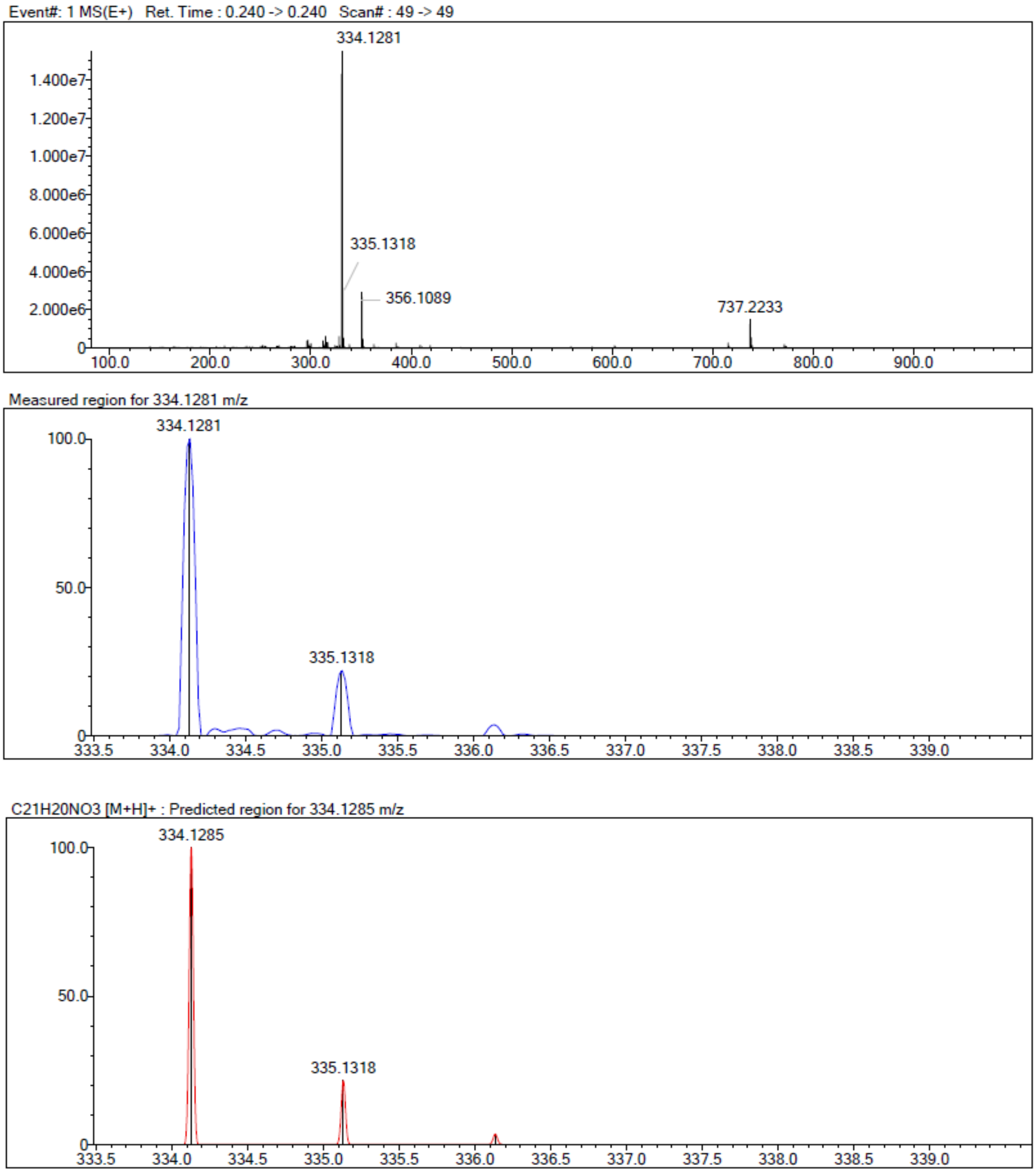

Rank	Score	Formula (M)	Ion	Meas. m/z	Pred. m/z	Df. (mDa)	Df. (ppm)	Iso	DBE
1	88.49	C21H20NO3	[M+H]+	334.1281	334.1285	-0.4	-1.12	88.76	11.0

19b

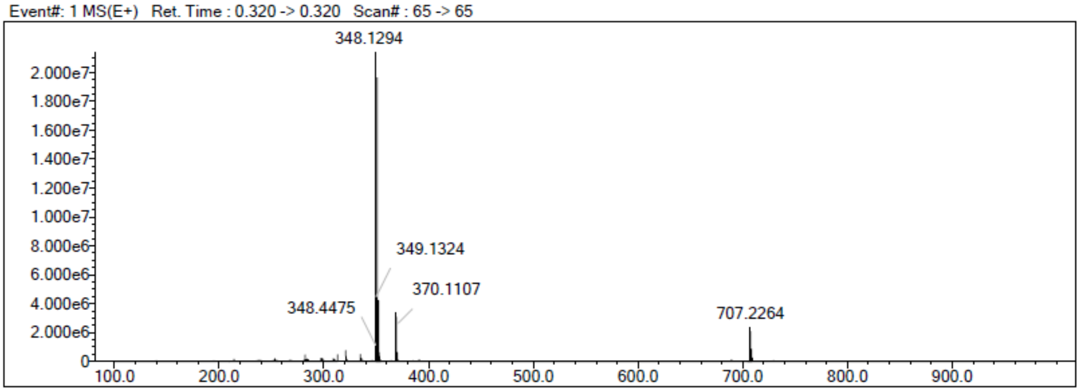

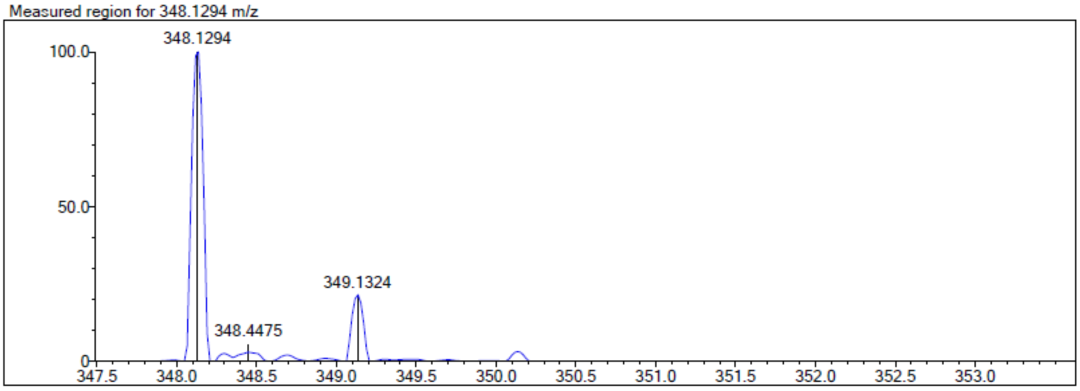

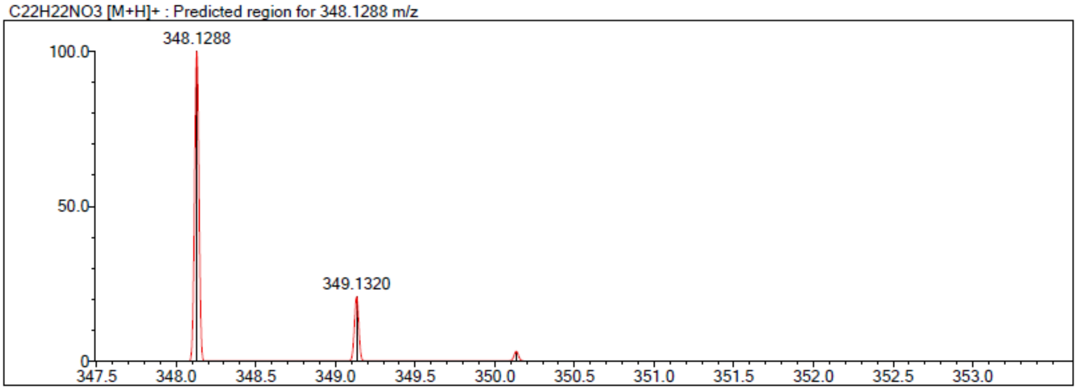

Rank	Score	Formula (M)	Ion	Meas. m/z	Pred. m/z	Df. (mDa)	Df. (ppm)	Iso	DBE
1	83.29	C22H22NO3	[M+H]+	348.1294	348.1288	0.6	1.75	84.88	11.0

21a

Event#: 1 MS(E+) Ret. Time : 2.860 -> 2.860 Scan# : 573 -> 573

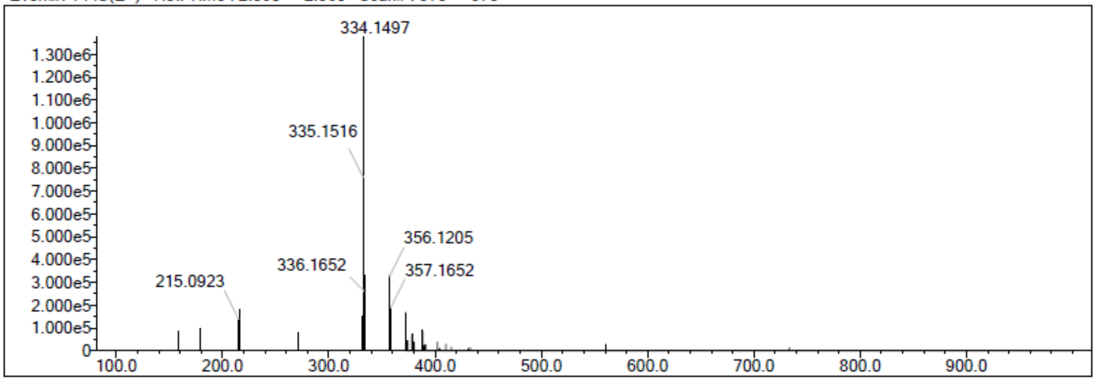

Measured region for 334.1497 m/z

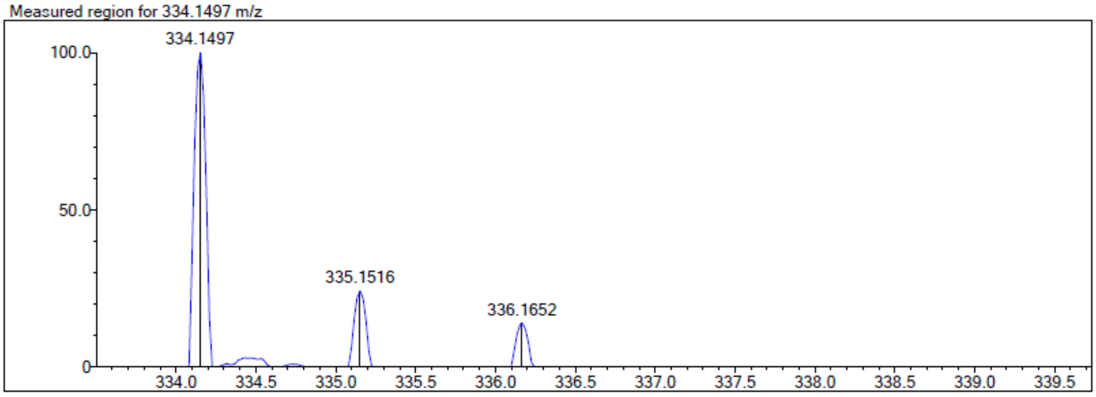

C21H20NO3 [M+H]+ : Predicted region for 334.1492 m/z

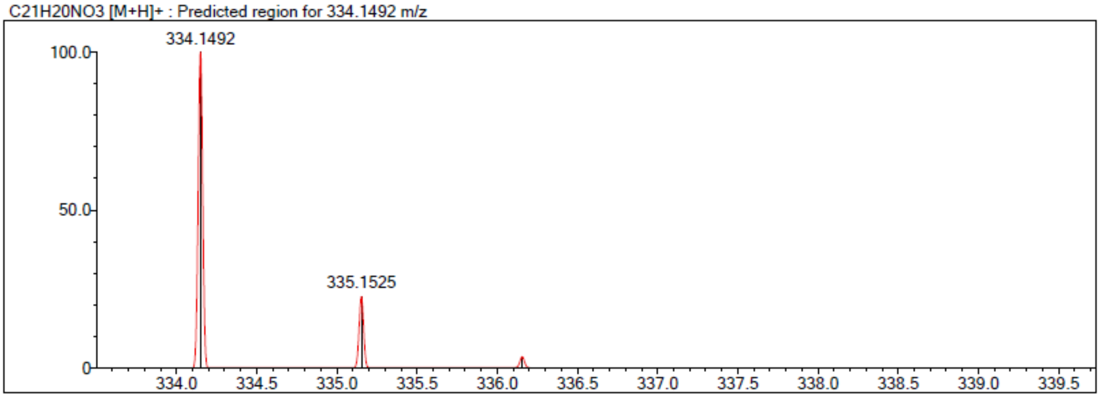

Rank	Score	Formula (M)	Ion	Meas. m/z	Pred. m/z	Df. (mDa)	Df. (ppm)	Iso	DBE
3	46.15	C21H20NO3	[M+H]+	334.1497	334.1492	0.5	1.40	46.61	11.0

21b

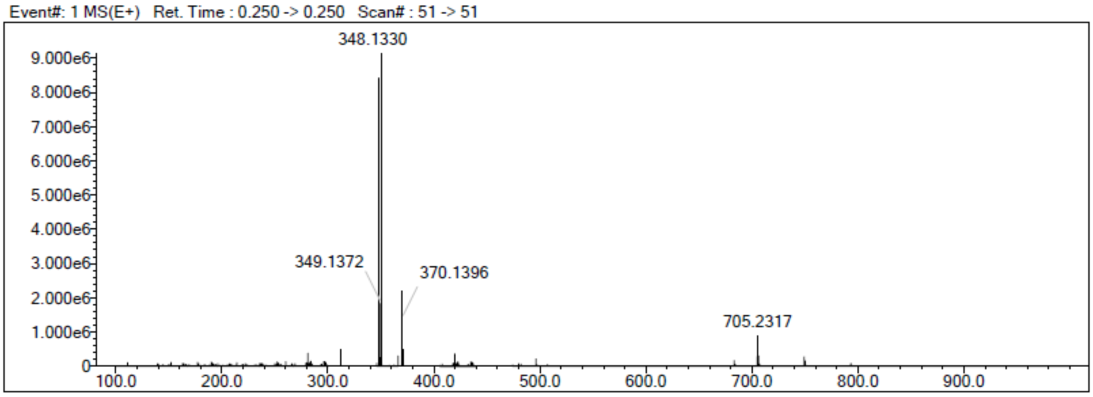

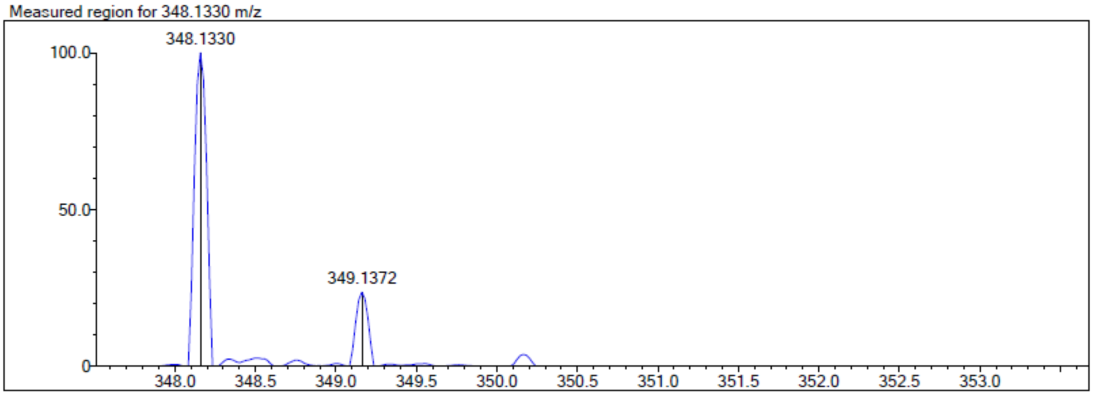

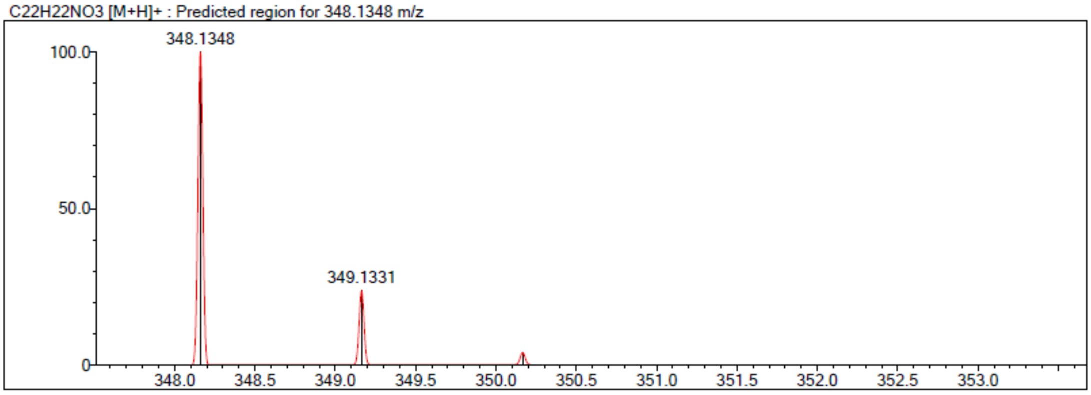

Rank	Score	Formula (M)	Ion	Meas. m/z	Pred. m/z	Df. (mDa)	Df. (ppm)	Iso	DBE
2	77.65	C22H22NO3	[M+H]+	348.1330	348.1348	-1.8	-3.11	81.98	11.0

S Ⅳ. Biological evaluation methods

S1. CCK-8 assay

The cells grown in logarithmical phase were seeded in 96-well plates with the density of 5 × 10^3/well. After 24 h, the medium containing different concentrations of tested compounds were added for another 48 h. Then, CCK-8 reagents were added to cells and continued to culture for 2 h. The optical density was recorded with a microplate reader at 570 nm (Thermo Scientific, Varioskan LUX). All the experiments were repeated independently at least three times and the IC_{50} values were calculated using GraphPad Prism 8.

S2. In vitro tubulin polymerization inhibition assay

The in vitro tubulin polymerization inhibition assay was conducted according to the manufacturer's instruction (cytoskeleton, cat. no. BK011P). Briefly, 5 μL of the tested compounds at the indicated concentrations which were prepared with the buffer solution (containing 80.0 mM piperazine-N,N-bis(2-ethanesulfonic acid) sequiso-dium salt (pH 6.9), 10.2% glycerol, 2.0 mM $MgCl_2$, 1 mM GTP, and 0.5 mM EGTA) was added to the plate. After the mixture was warmed to 37 °C for 1 min, the reaction was initiated by the addition of 55 μL of the tubulin solution which was kept on ice in prior. The fluorescence intensity enhancement (emission wavelength is 410 nm, excitation wavelength is 340 nm) was recorded every 60 s for 60 min in a multifunction microplate reader (Molecular Devices, Flex Station 3). The area under the curve was used to determine the concentration that inhibited tubulin polymerization by 50% (IC_{50}), which was calculated with GraphPad Prism Software version 8.02 (Graph- Pad Inc., La Jolla, CA, USA).

S3. molecular docking study

Virtual molecular docking of α,β-tubulin (PDB code: 1SA0) with different inhibitors were executed using MOE2012. Among the several reported crystal structures of colchicine domain, α,β-tubulin protein was first prepared by removal of the stathmin-like domain, subunits C and D, needless water molecules and colchicine and addition of hydrogen atoms. Colchicine binding site on the α, β-tubulin was defined as the site of ligand binding over α,β-tubulin. Structure files of the ligand were prepared for molecular docking by defining the number of torsion angles and addition of hydrogen atoms. To validate the feasibility of the MOE program, the docking studies were first performed on the reference compound colchicine. MOE successfully reproduced the binding conformations reported in the X-ray structure which was evidenced by the acceptable rootmean- square deviation (rmsd < 1 Å) of the atom coordinates. All structural images were obtained using PyMOL.

S4. Intracellular microtubule morphology detection

Immunofluorescence assay was applied to detect the intracellular morphology of microtubules. Briefly, the A549 cells samples which was treated with or without compound **14b** (10, 20, 50 nM) for 12 h was fixed in 4% prewarmed (37 °C) paraformaldehyde for 15 min, permeabilized with 0.5% Triton X-100 for 15 min and blocked for 30 min in 10% goat serum in sequence. Then, the cells were incubated with mouse anti-tubulin antibody (CST, USA) at 4 °C overnight. After the samples incubated with goat antimouse IgG/Alexa-Fluor 488 antibody (Invitrogen, USA) for 1 h, the nuclei were stained with Hoechst 33342 (Sigma, USA) in the dark at room temperature for 30 min, and then immediately visualized on a Zeiss LSM 570 laser scanning confocal microscope (Carl Zeiss, Germany).

S5. Cell cycle and apoptosis assay

After treated with or without compound **14b** (10, 20, 50 nM) for 24 or 48 h, the A549 cells were were detached by trypsin and harvested by centrifugation. For cell cycle analysis, the harvested cell

samples were fixed in ice-cold 70% ethanol overnight and removed with ice-cold PBS for three times. After treated with RNase A (Keygen Biotech, China) at 37 °C for 30 min, the cell samples were incubated with the DNAstaining solution propidium iodide (PI) (Keygen Biotech, China) at 4 °C for 30 min for subsequent analysis. For apoptotic detection, the cell samples were incubated with 5 μL of Annexin-V/FITC (Keygen Biotech, China) in binding buffer (10 mM HEPES, 140 mM NaCl, and 2.5 mM CaCl2 at pH 7.4) at room temperature for 15 min and PI solution for another 10 min. After the incubation, the prepared cell samples were immediately analyzed. Almost 10,000 events were detected by flow cytometry (Beckman Coulter, CytoFlex LX). The data regarding the number of cells in different phases of the cell cycle were or apoptotic cells were analyzed by CytExpert software.

S6 Caspase-3 activity evaluation

Caspase-3 activity was measured according to the manufacturer's instructions using a GreenNuc™ Caspase-3 assay kit for live cells (Beyotime, China). Briefly, after treated with or without compound **14b** (10, 20, 50 nM) for 48 h, the A549 cells were incubated with GreenNuc™ Caspase-3 Substrate (5 μM) in the dark for 15 min. After incubation, the fluorescence intensity (excitation wavelength: 485 nm, emission wavelength: 515 nm) was recorded using a multifunctional microplate reader (Thermo Scientific, Varioskan LUX).

S7 Intracellular ROS and MMP detection

Intracellular ROS and MMP detection were performed according to the manufacturer's instructions (Beyotime, China) using DCFH-DA and JC-1 probe, respectively. Firstly, the A549 cells were treated with or without compound **14b** (10, 20, 50 nM) for 12 h. For ROS detection and MMP detection, the treated cells were incubated with DCFHDA (10 μM) or JC-1 solution at 37 °C for 30 min, respectively. After incubation, the cells were washed with PBS and the nuclei were stained with Hoechst 33342 (Sigma, USA) in the dark at room temperature for 30 min, and then immediately visualized on a Zeiss LSM 570 laser scanning confocal microscope (Carl Zeiss, Germany).
